# Evidence for Water-Tuned Structural Differences in Proteins: An Approach Emphasizing Variations in Local Hydrophilicity

**DOI:** 10.1371/journal.pone.0045681

**Published:** 2012-09-25

**Authors:** Yasar Akdogan, Jörg Reichenwallner, Dariush Hinderberger

**Affiliations:** 1 Max Planck Institute for Polymer Research, Mainz, Germany; 2 Institut für Pharmazie und Biochemie, Johannes Gutenberg-Universität Mainz, Mainz, Germany; University of South Florida College of Medicine, United States of America

## Abstract

We present experimental evidence for the significant effect that water can have on the functional structure of proteins in solution. Human (HSA) and Bovine Serum Albumin (BSA) have an amino acid sequence identity of 75.52% and are chosen as model proteins. We employ EPR-based nanoscale distance measurements using double electron-electron resonance (DEER) spectroscopy and both albumins loaded with long chain fatty acids (FAs) in solution to globally (yet indirectly) characterize the tertiary protein structures from the bound ligands’ points of view. The complete primary structures and crystal structures of HSA and as of recently also BSA are available. We complement the picture as we have recently determined the DEER-derived solution structure of HSA and here present the corresponding BSA solution structure. The characteristic asymmetric FA distribution in the crystal structure of HSA can surprisingly be observed by DEER in BSA in solution. This indicates that the BSA conformational ensemble in solution seems to be narrow and close to the crystal structure of HSA. In contrast, for HSA in solution a much more symmetric FA distribution was found. Thus, conformational adaptability and flexibility dominate in the HSA solution structure while BSA seems to lack these properties. We further show that differences in amino acid hydropathies of specific structural regions in both proteins can be used to correlate the observed difference in the global (tertiary) solution structures with the differences on the primary structure level.

## Introduction

Since most biochemical processes occur in aqueous environments [1], the question how water interacts with biomacromolecules at a molecular level has been a long standing issue in the biological sciences. This was in particular fueled by the work of Kirkwood [2] and Kauzmann [3]. While thermodynamic functions themselves only unravel global views on the problem, they do not at all describe local properties such as density, flexibilities, composition and solvation effects at the level of solvent-solute interfaces [4]. Recently, several quite promising efforts have been undertaken to elucidate such physicochemical interactions between solvent and macromolecules on the nanoscale [4]–[10].

In protein research, serum albumin, partly due to its high abundance in the plasma of humans and other mammals, has been and still is a model protein, also for the study of protein-solvent interactions [11]–[13]. Serum albumin is the major transporter for low polar metabolites and drugs in the blood [11], [14]. Therefore, beyond its status as a model protein, it is important to understand the molecular mechanisms that enable albumin to transport several compounds in different organisms.

**Figure 1:**
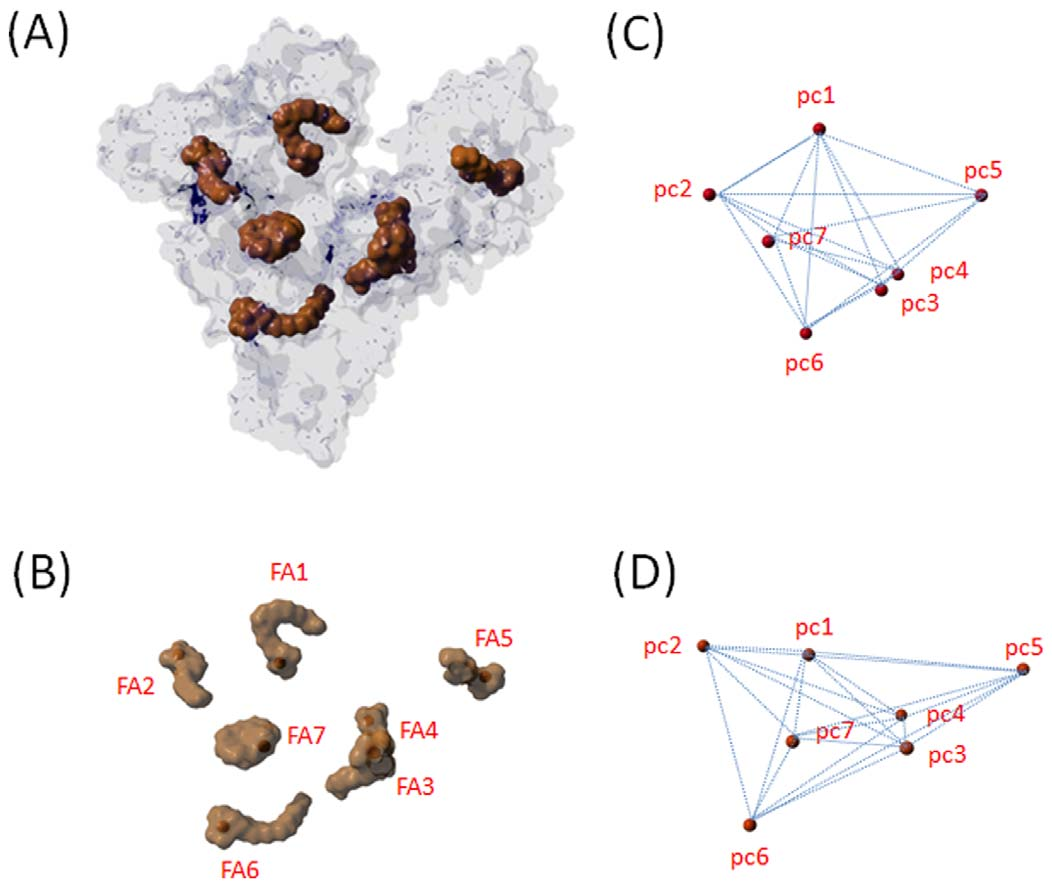
Location of fatty acids and their paramagnetic centers. Overview of the occupation and location of binding pockets of HSA with seven 5-DSA and 16-DSA ligands (PDB ID: 1e7i [27]). (*A*) congruent molecular surface area of HSA (bright blue) and FA_i_s (brown). (*B*) FA_i_s with according paramagnetic centers of 16-DSA (pc_i_) in red. (*C*) paramagnetic centers (pc_i_) of 5-DSA with their ensuing 21 possible interspin distances indicated (blue lines). (*D*) paramagnetic centers (pc_i_) of 16-DSA with their ensuing 21 possible interspin distances indicated (blue lines).

Here, we report differences in tertiary structures and conformational flexibilities of two different albumins and aim at elucidating the molecular (and physical) origin for these differences. Structural flexibility seems to constitute a key role in the ligand binding of albumin, e.g. as found by Trivedi et al. [15]. In previous studies, we probed the functional structure of human serum albumin (HSA) with respect to binding of fatty acids (FAs) [16]–[19] and a copper porphyrin [20] by methods of electron paramagnetic resonance (EPR) spectroscopy. This was achieved using self-assembled systems of HSA and spin-labeled FAs (see Fig. 1), namely DOXYL stearic acids (DSA) labeled with a paramagnetic DOXYL group either at the C5-position (5-DSA) or the C16-position (16-DSA) along the FA chain (Figure S1).

EPR spectroscopy has become increasingly popular in structural biology in recent years as it has been predicted from Hubbell and Altenbach, in particular with the development of nanoscale distance measurements using a pulse EPR method, double electron-electron resonance (DEER) [21]–[26]. In DEER spectroscopy, the r^3^-dependence (r = inter-spin distance) of the magnetic dipolar (through space) coupling between two electron spins is utilized to obtain the prevalent distance distribution and in principle orientational information.

Simple, conventional continuous wave (CW) EPR spectroscopy allows monitoring the uptake of the spin-labeled FAs by the proteins. DEER measurements give the spatial distribution of the ionic anchor points of FAs inside the binding channels (probed by 5-DSA) and the entry points into the FA binding channels (probed by 16-DSA) on the protein surface. This is indicated in Fig. 1 and has been worked out in Junk et al. [16] explicitly. Since the crystal structure of HSA complexed with seven long chain fatty acids has already been reported [27] (Fig. 1), HSA is a good model system to be compared with its functional solution structure. Note that similarly Calmodulin and Troponin C were studied with x-ray solution scattering by Heidorn and Trewhella [28]. Our distance measurements between the spin-labeled FAs revealed that the functional solution structure of HSA is largely in agreement with the crystal structure when probed with 5-DSA (ionic anchor points) but has a much more symmetric distribution of entry points to the binding channels (as probed with 16-DSA) than expected from the crystal structure [16]. This led to the conclusion that the inner part of the protein is rather rigid and may well be presented by the crystal structure, while HSA’s surface seems to be much more conformationally adaptive and flexible in solution.

We now extend our studies on HSA by examining FA binding to bovine serum albumin (BSA), and we illustrate the differences in FA uptake and the respective albumin’s solution structure. We aim at understanding the origin of the differences that we find in the functional solution structures by tracing them back to differences in local conformational adaptability and flexibility that ensue from their differences in the primary structures. Small changes in the amino acid sequences that can have tremendous effects on protein structure have been reported by inserting artificial mutations [29] or epigenetic amino acid exchanges [30] and in many cases it is the difference in hydrophilicity/hydrophobicity that drives such changes [6], [31], [32]. Hence, we in particular scrutinize the potential interactions with water and its H-bonding network to understand the different conformational arrangements.

## Materials and Methods

### Materials

Nondenatured HSA (>95%, CALBIOCHEM, Darmstadt, Germany), nondenaturated BSA (SIGMA-ALDRICH, Taufkirchen, München), spin-labeled FAs, 5- and 16-DSA (Sigma-Aldrich), and 87 wt % glycerol (FLUKA) were used as received. The spin-labeled FAs were partially reduced to EPR inactive hydroxylamines [16] (rDSA) by addition of phenylhydrazine (97%, SIGMA-ALDRICH) (see Fig. S1).

### Sample Preparation

Aqueous solutions of 2 mM HSA in 0.11 M phosphate buffer (pH = 7.2) and 26 mM DSA and rDSA in 0.1 M KOH were prepared. The combined concentration of DSA and rDSA in final buffered solutions of pH 7.4 was kept constant at 1.5 mM. The molar ratios of DSA and rDSA per BSA molecule were varied as 2∶0, 2∶2 and 2∶5. The final aqueous solutions of BSA/FA were supplied with 20% (v/v) of glycerol to prevent crystallization upon freezing. No changes in the CW EPR spectra were observed upon addition of glycerol. About 100 µL of the final solutions were filled into 3 mm (outer diameter) quartz tubes and shock-frozen in liquid-nitrogen-cooled isopentane for DEER measurements.

### EPR Measurements

A Miniscope MS200 (MAGNETTECH, Berlin, Germany) benchtop spectrometer was used for X-band CW EPR measurements at a microwave frequency of 9.4 GHz. Measurements were performed at 298 K using a modulation amplitude of 0.05 mT. The microwave frequency was recorded with a frequency counter, model 2101 (RACAL-DANA, Neu-Isenburg, Germany).

The four pulse DEER sequence π/2(ν_obs_)–τ_1_–π(ν_obs_)–(τ_1_+t)(ν_pump_)–(τ_2_– t)–π(ν_obs_)–τ_2_–echo was used to obtain dipolar time evolution data at X–band frequencies (9.2 to 9.4 GHz) with a BRUKER Elexsys 580 spectrometer equipped with a Bruker Flexline split–ring resonator ER4118X_MS3. The dipolar evolution time t was varied, whereas τ_2_ and τ_1_ were kept constant. Proton modulation was averaged by addition of eight time traces of variable τ_1_, starting with τ_1,0_ = 200 ns and incrementing by Δτ_1_ = 8 ns. The resonator was overcoupled to Q ≈ 100.

The pump frequency, ν_pump_, was set to the maximum of the EPR spectrum. The observer frequency, ν_obs_, was set to ν_pump_ = +61.6 MHz, coinciding with the low field local maximum of the nitroxide spectrum. The observer pulse lengths were 32 ns for both π/2 and π pulses, and the pump pulse length was 12 ns. The temperature was set to 50 K by cooling with a closed cycle cryostat (ARS AF204, customized for pulse EPR, ARS, Macungie, PA). The raw time domain DEER data were processed with the program package DeerAnalysis2008 [26]. Intermolecular contributions were removed by division by an exponential decay with a fractal dimension of d = 3.8. As shown in a previous study, the deviation from d = 3.0 originates from excluded volume effects due to the size of the protein [16].

### Structural Analysis

Visualization, Molecular Modeling, and structural alignments of the proteins were carried out using YASARA Structure Software [33].

## Results

**Figure 2:**
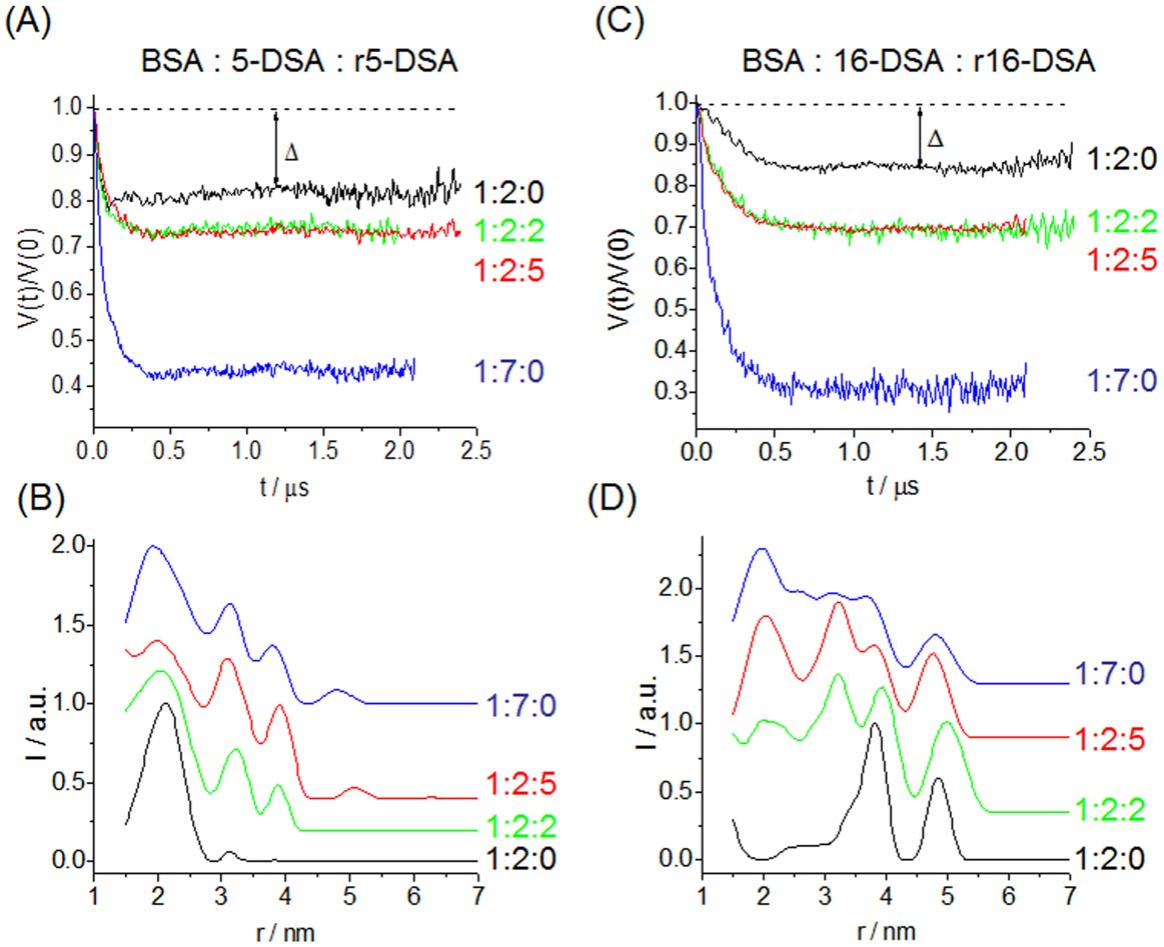
Intramolecular part of the DEER time-domain data. Extracted distance distributions of 5-DSA (*A*, *B*) and 16-DSA (*C*, *D*) in BSA solutions with varying numbers of paramagnetic and diamagnetic FAs (the numbers denote: Albumin : DSA : rDSA). Δ indicates the modulation depths.

**Figure 3:**
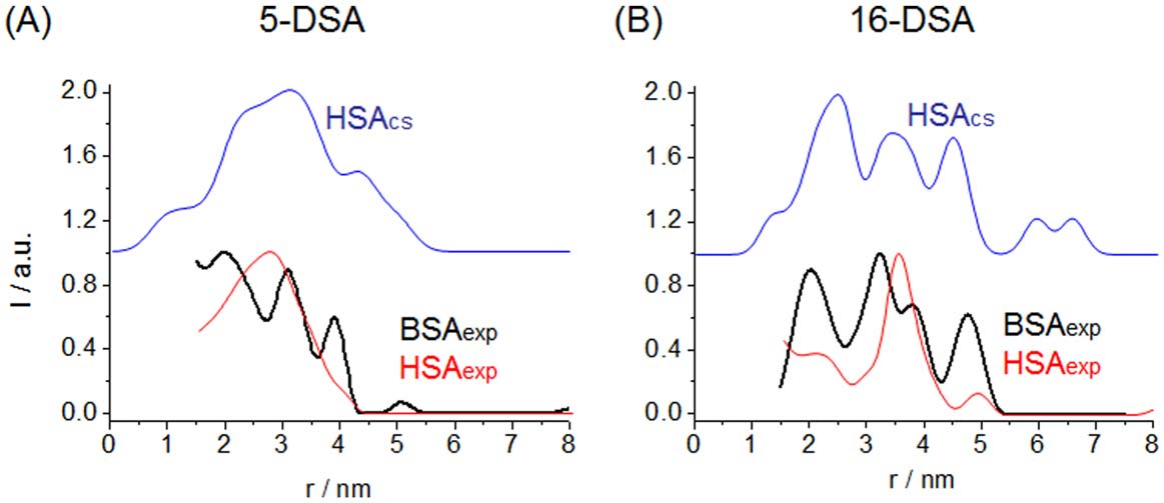
Comparison of the distance distributions of fatty acids. 5-DSA (*A*) and 16-DSA (*B*) obtained from DEER measurements in HSA (red, 1∶2:4) and in BSA (black, 1∶2:5) solutions with the calculated distributions from the crystal structure of HSA (blue) assuming that all seven binding sites are occupied by FAs (Albumin:DSA:rDSA).

Throughout the article, we compare the BSA-related findings with our previously published EPR measurements on HSA and the crystal structure-derived data, where applicable. All continuous wave (CW) EPR results of DSAs in BSA and in HSA are displayed in the Supporting Information (Fig. S2 and S3). These CW EPR measurements prove that BSA rigidly binds as many FAs as HSA. When studying the occupation of the binding channels, one has to avoid multispin interactions that arise when three or more spin labels interact simultaneously in a single protein [18], [34]. To this end, paramagnetic and diamagnetic FAs (rDSA), are added simultaneously to obtain higher FA loadings in the protein while keeping the average number of EPR-active FAs at two. In Fig. 1 we additionally show how to construct the theoretical distance distributions of the paramagnetic centers of the seven bound fatty acids in the crystal structure. The experimental distance distributions (shown in Fig. 2) are compared with these theoretical distributions in Fig. 3.

The immobilized 5-DSA species reports from an inside view of the protein and 16-DSA reports from the binding site entry points and thus from the protein’s surface (see also A_zz_–differences in Fig. S2 and S3). Further details can be found in Junk et al. [16].

For HSA, these spin-diluted systems have shown almost identical DEER distance distributions regardless of the overall number of FAs added [16]. However, after addition of two spin labeled 5-DSA and additional rDSA to BSA, distinct new distances are obtained at about 3.2 nm and 3.9 nm that were not observed in HSA (Fig. 2 *B*). Finally, a rather broad, asymmetric [16] distribution of 5-DSA can be observed in BSA covering a range similar to that of 5-DSA in HSA with the exception that in BSA more distinct peaks are observed, as shown in Fig. 3 *A*. Although the new signals are obtained in BSA upon addition of more FAs, this does not indicate consecutive filling of individual sites in BSA. The CW EPR spectra are identical regardless of the loading order of paramagnetic and diamagnetic FAs.

The DEER measurements of the system BSA and 16-DSA show dominant peaks in the distance distributions in Fig. 2 *D*. They are located at 3.82 nm and 4.85 nm, and in the corresponding HSA crystal structure the distances are 3.83 nm (distance between sites 2–6), 3.70 nm (sites 4–6) and 4.64 nm (sites 2–3) [27]. For 5-DSA and BSA, the dominant peak is centered at 2.13 nm and the corresponding sites are 2.14 nm (sites 4–5), 2.18 nm (sites 4–6) and 2.18 nm (sites 6–7). These analyses show that FAs are predominantly located at binding sites of 6 and 4, but also other binding sites 2, 3, 5, 7 are filled at initial loading. This suggests that at our concentrations used (∼1 mM) the FA binding to BSA is not strictly consecutive but already at low FA to protein ratios (2∶1) rather all sites are more or less occupied to a certain degree, just as it was observed for HSA.

Remarkably, when r16-DSA is added to BSA in solution, distances at about 2.0 nm and 3.3 nm are found for 16-DSA (ratio 1∶2:5 HSA/DSA/rDSA) that were not present in the HSA solution (Figs. 2 *D* (red curve) as well as 3 *B* (black curve)). Fig. 2 also shows the DEER data and distance distributions when BSA is loaded with exclusively 7 paramagnetic 5-DSA or 16-DSA (ratio 1∶7:0). This shows that there is no principal difference between the spin-diluted and the fully paramagnetic systems, only the peaks are much narrower in the spin-diluted systems as they are devoid of the above mentioned multi-spin effects [18] (for further insights consult the Supporting Information S1).

The distance distributions of FAs in BSA and in HSA solutions obtained from DEER measurements are additionally compared with distributions determined from the crystal structure of HSA co-crystallized with seven stearic acids (pdb-ID: 1e7i) [27] in Fig. 3. The distances between the C-5 and C-16 atoms of 5-DSA and 16-DSA in all seven FA binding sites of HSA (denoted HSA_cs_) were obtained from this crystal structure as is shown in Figs. 1 *C* and *D* [16].

For 5-DSA, a broad distance distribution reveals a highly asymmetric [16] distribution of the C5-positions of the FAs in HSA and BSA. These DEER-derived distributions of 5-DSA in BSA (Fig. 2 *B* and 3 *A*) are largely and principally in agreement with the distance distribution expected from the HSA crystal structure. As the 5-position of most fatty acids [27] probes the region in the binding channels close to the mostly deeply buried ionic anchor groups, one can conclude that in HSA and BSA the inner part of the proteins can be viewed as being rather rigid and inflexible (in agreement with the HSA crystal structure).

In contrast, the DEER distance distributions of 16-DSA in BSA and in HSA are clearly different. Surprisingly, the DEER-derived distribution of 16-DSA in BSA is in better agreement with the HSA crystal structure-derived distribution than the respective DEER data of HSA. The three characteristic peaks at 2.5, 3.5, and 4.5 nm in the HSA crystal structure are matched experimentally in BSA in solution (at 2.0, 3.3, and 4.7 nm).

In general, the comparison of 5-DSA distance distributions yields lower RMSD values (Table S3), substantiating that in case of 5-DSA (and hence close to the FA anchor groups) the experimental DEER and the crystal structure distributions are in rather good agreement. The two experimental (red and black in Fig. 3 *A*) curves, HSA_exp_ and BSA_exp_, in fact give the lowest RMSD of 0.171 as shown in Table S3. For the 16-DSA distance distributions (Fig. 3 *B*) the best agreement was found between HSA_cs_ (blue) and BSA_exp_ (black) with an RMSD of 0.201. This is remarkable, as the lowest similarity was found between HSA_exp_ (red) and the HSA crystal structure, HSA_cs_ (blue), with a value of 0.317. These findings quantitatively reinforce the following: Assuming that the number and principal location of FA binding channels in the protein is identical in HSA and BSA (see Fig. S2 and S3), this suggests a much more asymmetric distribution of the entry points (seen by 16-DSA) to FA binding channels in BSA. Based on the “coarse-grained” view of the protein structure given by 16-DSA (and partly 5-DSA) distance distributions in Fig. 3, the BSA solution structure (Fig. 3 *B*, black) may be seen as clearly resembling HSA in its crystalline state (Fig. 3 *B*, blue) while the HSA solution structure (Fig. 3 *B*, red) more strongly deviates from the HSA crystal structure.

It should be noted that to this date no crystal structure of BSA with FAs has been reported, although recently a first BSA dimer structure has been uploaded on the RCSB protein data base [35]. Thus, we always use the HSA crystal structure (pdb-ID: 1e7i) [27] for comparison, even with BSA solution data.

**Figure 4:**
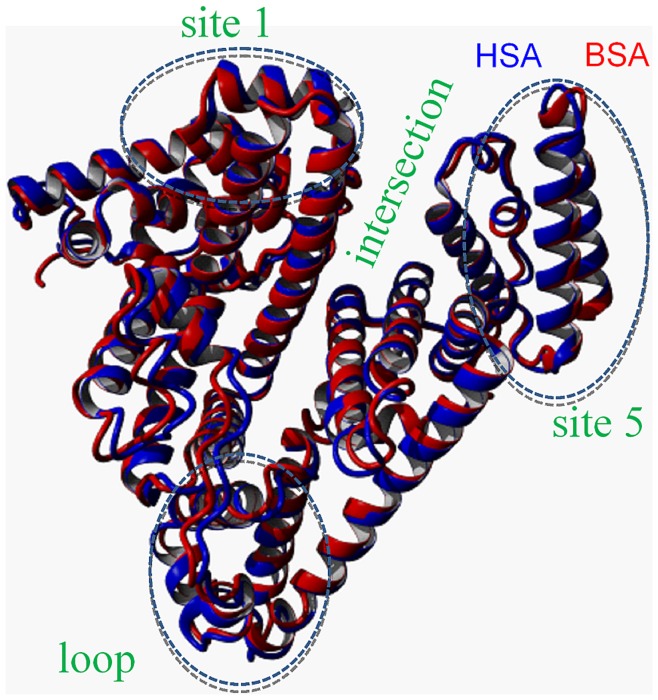
Crystal structure alignment of HSA and BSA. HSA without FAs (pdb-ID: 1BM0, blue) aligned with the crystal structure of BSA without FAs (pdb-ID: 3v03, red) using the MUSTANG algorithm. The regions of interest (site 1, site 5, intersection, loop) are highlighted in green.

To quantitatively compare structural identities in the crystal state, both proteins in their ligand-free form (pdb-IDs 3v03 [35] and 1BM0 [36]) were aligned with the MUSTANG algorithm [37] which delivers an RMSD of 1,361 Å and a sequence identity of 75,52% over 572 aligned residues. From these values one can assess that the aligned structures are in very good agreement (Fig. 4).

## Discussion

### Primary Structure Differences between HSA and BSA

We now focus on identifying the molecular origin of the DEER-derived differences between the solution structures of BSA and HSA. To this end, we scrutinize both proteins’ primary amino acid sequences, especially differences in their individual hydrophobicities. Since local thermodynamic quantities are key quantities of any study of biochemical processes in solution, hydrophilicity and hydrophobicity are highly important parameters describing interactions between solvent (for proteins in essence water) and solute [38]. The biochemical reasons for amino acid differences can be manifold, besides evolutionary differences e.g. availability of certain nutrients can play a role which was proposed to lead to an epigenetic formation of alloalbumins in between a single species [30].

Many different methods to assess hydrophobic regions in proteins are currently in use, e.g. the molecular hydrophobicity potential, MHP [31], [32]. We consulted four rather simple hydropathy scales of independent origin to obtain a quantitative analysis of differences in hydrophobicity/hydrophilicity of BSA and HSA. We confer to the scales of Engelman et al. (GES) [39], Eisenberg et al. (ES) [40], Naderi-Manesh et al. (NM) [41] and Kyte & Doolittle (KD) [42].

We have in detail compared cross correlations between these four scales in the Supporting Information S1 and found a linear dependence between the scales that can be quantified in terms of a Pearsońs r value, which in all cases is > ±0,85 (Table S1, Fig. S7). Thus all hydropathy scales are strongly correlated with each other, although having different theoretical and experimental foundations.

After proving that for all four hydropathy scales essentially and quantitatively lead to the same results and thus for the following discussion we mainly discuss the BSA-HSA differences in terms of the hydropathy scale of Kyte & Doolittle, which is the most commonly used and simplest, yet intuitive scale to characterize amino acids thermodynamically. Specifically, the Kyte & Doolittle scale hydropathy index (HI) describes the change in Gibbs energy when exposing an amino acid from a purely hydrophobic environment to water. Hence, negative hydropathy values denote polar and strongly hydrogen-bonding amino acids, while positive values stand for hydrophobic amino acids. We compared each congruent amino acid of HSA and BSA (i.e. the window range according to Kyte and Doolittle equals 1) to get a resulting net hydropathy index difference ΔHI by subtracting the corresponding values (see Eq. S1). This allows focusing on the differences and additionally reduces noisy scales. Positive ΔHI values can be interpreted as a hydrophobic and negative values as a hydrophilic shift in HSA. Where appropriate, we also compare the actual amino acids and their hydropathies one by one (Fig. S5–S6). Note that the KD hydropathy scale is often used for membrane-bound proteins [42], [43] with window ranges of 7 up to 20 amino acids.

We explicitly examine individual residues and do not average the hydropathies by using larger windows. A detailed explanation of this treatment can be found in the Supporting Information S1.

Note that we here simply try to correlate the hydropathy differences and the positions in the crystal structure with the observed discrepancies in our solution structures.

In general, HSA (overall hydropathy Ω_HSA_ = −230.8, see Eq. S2) has an excess of 48.4 hydropathy points compared to BSA (Ω_BSA_ = −279.2) and thus HSA should altogether be more hydrophobic. This tendency can be confirmed by all other 3 scales (excess hydropathy points of HSA: GES: 9.4; ES: 16,8; NM: 34,8) when they are normalized to KD (Table S2). Remarkably, when inspecting the residue positions that are different, it becomes obvious that these deviations are not homogeneously spread throughout the full sequence but occur rather clustered (see Fig. S4). A similar observation was also made by Billeter et al. [44] when comparing prion protein structures of different mammalian species.

Many of the albumin differences are even at residues that are surface-exposed, which is important when keeping in mind that HSA can be considered less hydrophilic than BSA as evidenced by their Ω_x_. Without claiming completeness, we have explicitly identified four regions that are of high interest, structure- and function-wise, that show an accumulation of amino acid differences and ΔHI extrema between BSA and HSA: the intersection between subdomains IB and IIIA located prominently in the in center of the protein, a surface-exposed loop region in subdomain IIB, and two FA binding sites usually referred to as site 2,4 and 5 [45]. These regions are indicated in Fig. 4. Interestingly, Majorek et al. [35] found very similar regions during identification of antibody epitopes for immunological studies of bovine (BSA), equine (ESA) and rabbit serum albumin (RSA). It is thus not farfetched to suppose that those regions are of special functionality in combination with water environment for any ligand which has to be bound to albumin.

**Figure 5:**
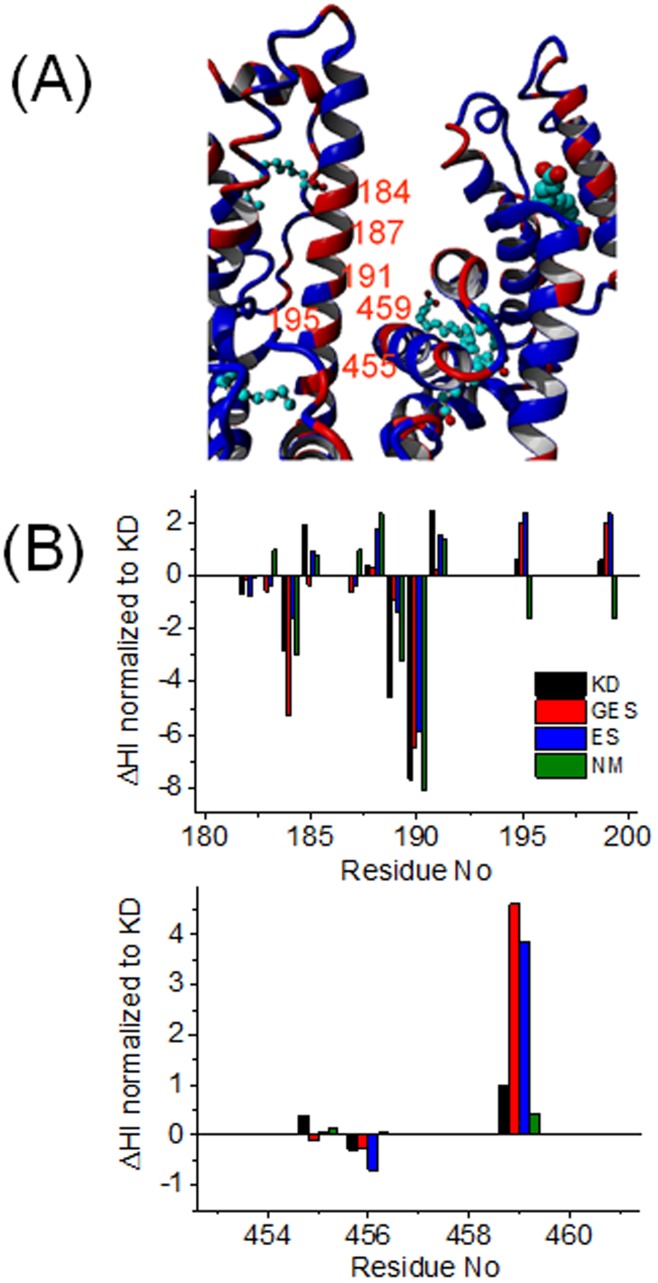
Intersection region. (*A*) Intersection region between subdomains IB and IIIA of HSA with bound stearic acid (pdb-ID: 1e7i). Identical amino acids in HSA and BSA are blue, differing amino acids are red. (*B*) Plot of ΔHI for residues 180–200 and for 452–460.

**Figure 6:**
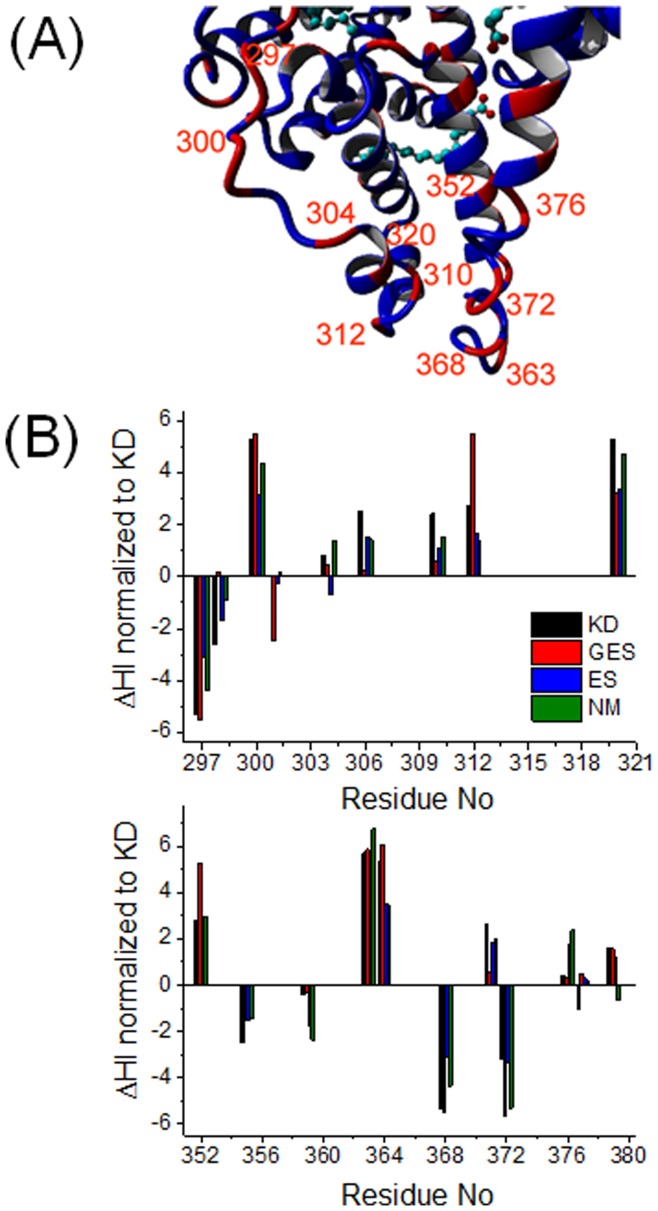
Loop region. (*A*) Subdomain IIB of HSA with bound stearic acids (pdb-ID: 1e7i). Identical amino acids in HSA and BSA are blue, differing amino acids are red. (*B*) Plot of ΔHI for residues 297–320 and for 351–380.

**Figure 7:**
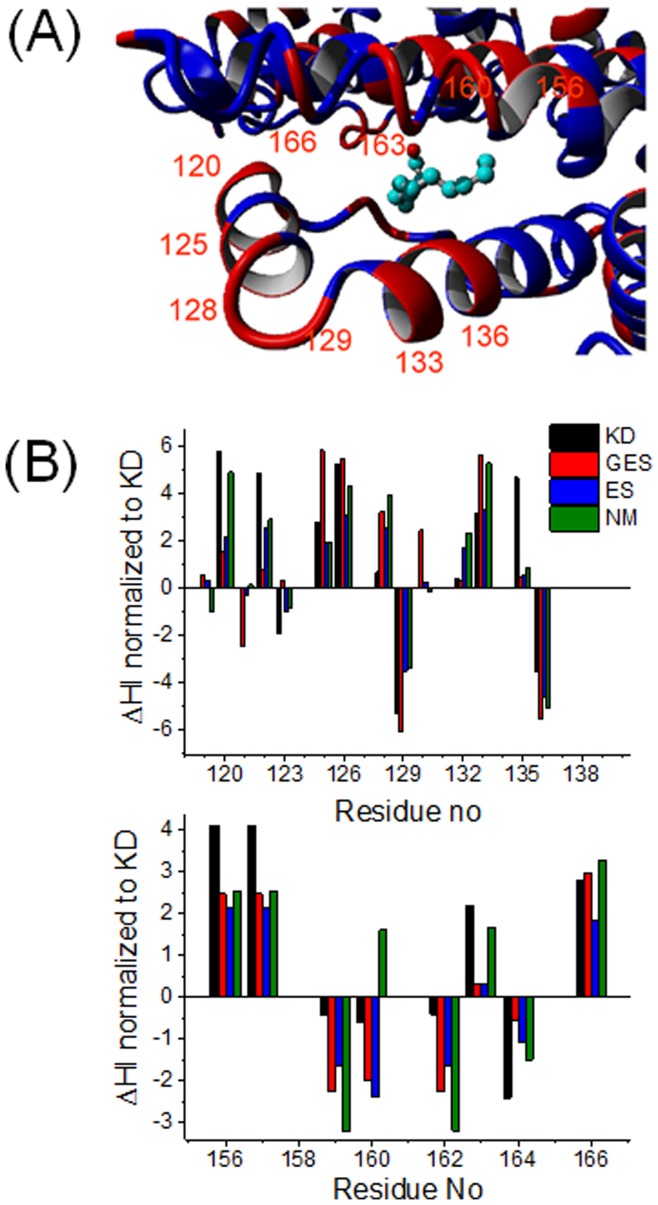
Site 1. (*A*) Site 1 in the subdomain IB in HSA with bound stearic acids (pdb-ID: 1e7i). Identical amino acids in HSA and BSA are blue, differing amino acids are red. (*B*) Plot of ΔHI for residues 119–136 and for 154–168.

**Figure 8:**
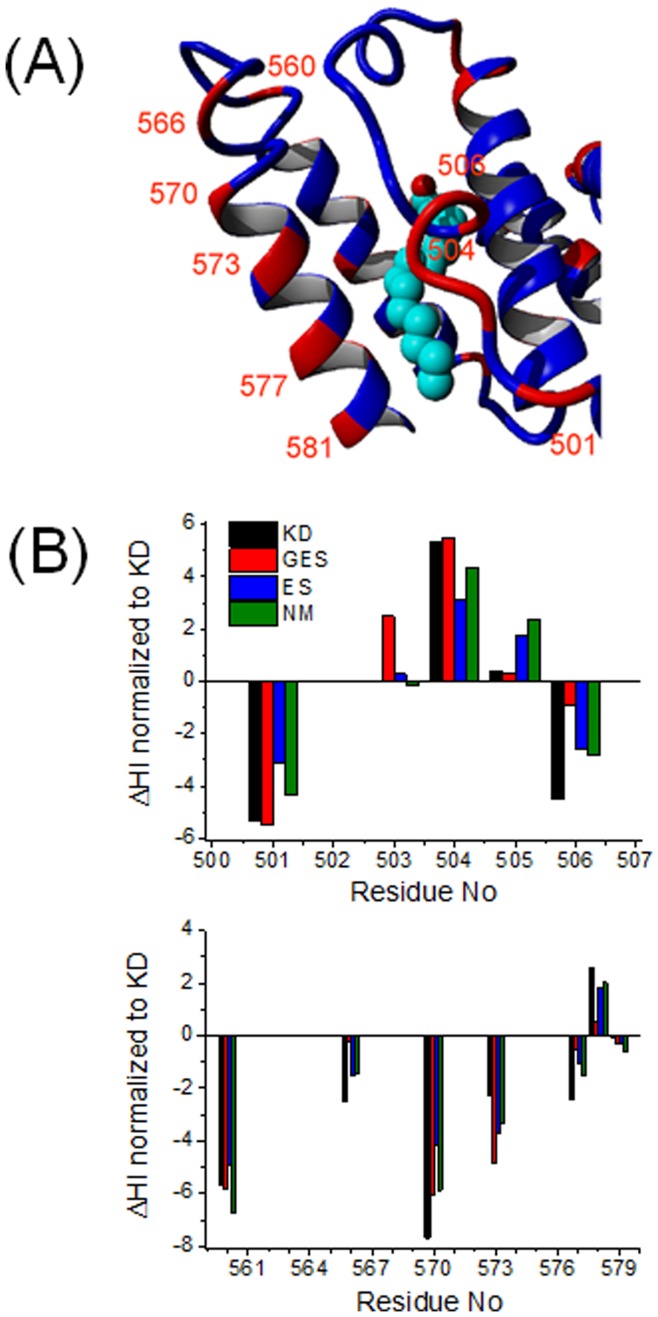
Site 5. (*A*) Site 5 in the subdomain of IIIB in HSA with bound stearic acid (pdb-ID: 1e7i). Identical amino acids in HSA and BSA are blue, differing amino acids are red. (*B*) Plot of ΔHI for residues 498–509 and for 560–580.

We now further describe these regions and explicitly report the differences between BSA and HSA in terms of the KD hydropathy index. Note that in Figures 5–8 we nonetheless present the differences in hydropathy for all four hydropathy scales tested to make it clear that our discussion is not bound to the chosen hydropathy index. Fig. 5 shows the region between subdomains IB and IIIA which is solvent accessible. Except for one arginine, HSA and BSA differ in amino acid sequence throughout the helix between residues 182 and 191 in IB, albeit forming an equivalent helix array as Kabsch and Sander [46] have proposed. While residues 182, 185, 188, 189, 195 and 199 are not solvent accessible, residues 184, 190 and 191 are solvent-exposed and accessible by bulk water. The ΔHI between both proteins is found to be very strong in this region; HSA seems to have extraordinarily strong hydrophilic properties, while BSA is strongly hydrophobic at analogous residues 189 and 190.

Having a closer look, residue 190 (K = −3.9) in HSA corresponds to L = 3.8 in BSA. This indicates that these domains might have different flexibilities according to Lum-Chandler-Weeks (LCW-) theory [7]. Thereby their interactions with water clearly differ, which can be explained by a hydropathy lock immobilization of the two domains in BSA between hydrophobic residues 455 (L) and 456 (I) and the opposing 189 (V) and 190 (L) segments. In HSA, this region may simply still be water-accessible due to the strongly hydrophilic residues and can also be seen as indicating a “tuning” of the local solution structure topology with assistance of water. In addition, residue 189 is varied from G (−0.4) in HSA to more hydrophobic V (4.2) in BSA. Therefore, BSA could produce a drying out phase transition zone, which depletes the water density between both domains.

The sequence belonging to the helix ranging from 455 and 457 in domain III B also differs in composition, but in both proteins it can be considered as extremely hydrophobic. Altogether, given the prominent interdomain position, tuning flexibility and water interaction at this point may well trigger structural difference on a larger topology scale.

Another site of interest is found in subdomain IIB, where significant amino acid differences are found between BSA and HSA (see Fig. 6). A loop between 295 and 312 is solvent-exposed and residues 297 and 300 show large hydropathy deviations. HSA has a set of more hydrophobic residues between 300 and 320 compared to corresponding residues in BSA. The other significant albumin differences are in close proximity to this loop and are situated on the opposing solvent-exposed loop ranging from residue 360 to 376. There is a strong hydrophobic shift towards HSA at residues 363 and 364. Those amino acids are right at the tip pointing into the water bulk. By hypothetically exposing this region to water, one could imagine that it will flip inside the protein interior and alter the shape of the protein, potentially becoming slightly more globular.

Being connected to several helices in HSA, rearrangements in these prominently exposed loops that have a high degree of motional freedom may be envisioned to have significant impact on the tertiary structure. Note that very similar observations were made for calmodulin and troponin C [28] and such an argument is also underpinned by Kim et al. [47] who found by EPR methods that the effect of solutes on the conformational sampling of loops can lead to a more compact (globular) form of outer membrane transporters. In contrast to HSA, BSA (at least without fatty acids) very likely exposes this region to the water bulk, having a set of strongly hydrophilic residues from position 363 to 367 (K, D, D, P, H) and 311 to 323 on the opposing loop. An interesting view on opposing hydrophilic residues has been given by Ben Naim [4], who claims, that water molecules might stitch together two domains by forming double hydrogen-bonded bridges between them. Such a domain connection and a better inclusion of these loops in the water H-bonding network for BSA may together lead to a reduced conformational flexibility at this decisive point.

Fig. 7 shows one of the fatty acid binding sites, usually denoted as site 1. It is situated in the center of the four helix bundle of subdomain IB. This site is solvent accessible. The congruent amino acid positions in HSA are again more hydrophobic compared to BSA. More precisely, the significantly more hydrophobic residues 120, 122, 126, in HSA are all located on a small surface-exposed helix. Positions 156 and 157 are also prominently hydrophobic in HSA, while the following four residues from positions 159–162 are extremely hydrophilic in both proteins. Note that only two amino acids are identical in the range from 156–164 (Y and A, see SI Fig. S5 and S6). This might indicate a difference in how FAs are bound in the two proteins. The shape of the binding pocket may well be altered and the water exposition may be tuned similarly to the mechanism that can be inferred for subdomain IIB (Fig. 6).

In the case of fatty acid binding site 5 (see Fig. 8) the pattern observed so far that HSA is generally more hydrophobic at solvent-exposed residues is actually inverted.

HSA has strongly hydrophilic residues between 560 and 577 compared to those of BSA. Binding site 5 can be found in a hydrophobic channel of subdomain IIIB. Except for residue 579 all of the amino acids in this region are solvent exposed and residue 570 have a very strong ΔHI value of −7.7 between HSA (E = −3.5) and BSA (V = 4.2). It is located far away from the FA entry point but marks the transition from a helical to a loop region which may control the motion of the helices forming the FA entrance. Around binding site 5, both proteins may have clearly different solution conformations, as residue 560, which is located at the other end of this loop may contribute to form a hinge task in HSA. When comparing the HSA crystal structures 1e7i with 1BM0 (with and without FAs bound), it is site 5 that physically moves out by about half a nanometer when long chain FAs are bound [27].

The opposing side of the binding channel entry point is formed by a loop from residues 501 to 506, which are all surface-exposed. The ΔHIs change dramatically from residue 501 where a charged, strongly hydrophilic glutamic acid in HSA is matched by a neutral, slightly hydrophobic alanin in BSA (E_HSA_-A_BSA_ = −5.3) to residue 504 where this is inverted (A_HSA_-E_BSA_ = 5.3) and at residue 506 where a threonine in HSA with rather neutral HI corresponds to a strongly hydrophobic leucin in BSA (T_HSA_-L_BSA_ = −4,5). In HSA, this full series of amino acids alternates between hydrophilic and hydrophobic, while in BSA this loop is overall much more hydrophobic (see Fig. S5 *C*). Therefore, the interaction with water of the surface region of site 5 in HSA clearly deviates from that in BSA. For HSA, the helix region 570–580 is clearly more hydrophilic than for BSA, i.e. also better “anchored” into the H-bond network.

Speculating about the molecular mechanism of FA binding, the opposing loop region may play a crucial role by being flipped inside (closed) or outside, solvent exposed (open). BSA is clearly more hydrophobic in the loop region, too, while HSA again complements an interesting, alternating pattern of strongly hydrophilic and hydrophobic amino acids. This pattern – which can be assumed to neither have a strong energetic and entropic propensity to be exposed to water nor to be buried – may again translate into more conformational flexibility [48] and adaptability in HSA.

### Correlation of Differences in Primary Structures, Crystal Structures, and Solution Structures

Despite the similar primary structure of BSA and HSA, our experimental DEER results – to the best of our knowledge for the first time - show BSA to be rather rigid in solution and to clearly deviate in its functional structure from HSA on the nanoscale. We have identified four regions in both proteins (not necessarily complete) that feature severely different amino acids. While we cannot directly trace back the apparent difference between BSA and HSA solution structures to the difference in hydropathies, we can plausibly argue that the clustered amino acid differences may well lead to severe changes in the structure when exposed to water, as it had been observed for other proteins before [10], [28].

It is striking that the amino acid sequence and crystal structure similarities between HSA and BSA are reflected in the solution structure of BSA. In our “coarse-grained” view of the bound FAs, we find a distinct three peak pattern strongly resembling the distribution of FAs in the crystal structure of HSA. As no crystal structure containing FAs is currently available for BSA, one may conclude that the HSA crystal structure, which very likely describes a conformation close to the minimum of the potential energy surface, is a very good description for the energetic minimum in FA-loaded BSA, too. Based on our DEER results, one can plausibly speculate that in solution, HSA, in contrast to BSA, gains a much larger conformational flexibility and in the overall conformational ensemble of HSA a much more symmetric surface is exposed to potential ligands to be transported. BSA does not seem to have this flexibility at the surface, which may in fact affect protein function. It should be noted that although proteins with similar functions across different species may have similar crystal structures [49], their functional properties in solution may vary decisively. This was e.g. confirmed by recent investigations showing that water interacting with solutes can heavily affect functional properties [6]. Close proximity of water to extended surfaces with hydrophobic properties is energetically unfavorable because hydrogen bonding networks can no longer be maintained. As a result, the water density is depleted near the hydrophobic surface leading to a drying transition not least depending on length scale [6], [7], [38]. Furthermore, we have recently shown that even methyl groups of methanol in water-methanol mixtures surround small inorganic disulfonates such that they least disturb the hydrogen-bonding network [50].

Kabsch and Sander [46] explained very clearly that amino acid sequence homologies in proteins do not necessarily reveal functional relationships between them. Furthermore, sequence homologues do not necessarily have to feature identical conformations in their crystal structures. As can be seen from comparing the HSA and BSA structures (pdb-IDs 1BM0 and 3v03, respectively), 75.52% sequence homology can on the other hand produce almost identical 3-D crystal structures (see Fig. 4), which confirms that the 3-D structural homology threshold for proteins longer than 80 residues produces similar structures for already 25% sequence compliance [51]. These amino acid differences hence do not alter the energetically minimized state devoid of water very strongly. We have identified many of the amino acid sequence differences to be clustered at certain regions in the protein that may very strongly influence the FA binding (binding sites 1 and 5) or even the global structure (central interface between subdomains IB and IIIA and loop regions in subdomain IIB). For example, the apparent rigidity observed in BSA may be accredited in parts at least to the hydropathy “lock” in the prominent intersection region between subdomains IB and IIIA (see Fig. 5), while in HSA water may well be able to more deeply penetrate this intersection region. Furthermore, the loop region at the tip of subdomain IIB is so strongly water exposed and simultaneously bridges long helix regions that even small changes in hydrophobicity (HSA exposing several hydrophobic amino acids to water) may well increase structural agility and direct structural changes at seemingly remote regions by allosteric effects. From our DEER-derived measurements of the functional structures in solution, these differences hence seem to be rather correlated with specific modifications to tune the shape, flexibility, adaptability and binding capacities of a protein by interacting with the surrounding solvent molecules, and by influencing fluctuations in water density [5]–[7].

There is a very interesting recent nuclear magnetic resonance (NMR) approach from Nucci et al. [10] using NOE/ROE-ratios in NMR and reverse micelle encapsulation for monitoring protein hydration. The authors found that all over the protein surface there are regions of hydration clusters of strongly varying dynamics, which can be regarded as evidence for water taking a protagonist role in modifying protein functions such as shaping, folding, stability and dynamics. Using ^13^C-NMR, Marlow et al. [48] went one step further and connected the hydrophobic effect to the conformational entropy, which is supposed to play a key role on ligand binding. Our site-specific findings looking at local hydropathy values and coarse-grained (EPR-based) structural information arrives at similar insights. By comparing bromocresol green (BCG) binding affinities of different mammalian serum albumins [15] it was found that the exchange of only one amino acid in a binding pocket region, as shown with alloalbumins of rhesus macaque, could give a creature an evolutionary advantage [30], or decides between physiologically active proteins or their pathological amyloid fibril storage in tissue [52]–[54]. Phosphorescence depolarization was used to understand the relation between solution structure and crystal structure of HSA and BSA. It was found that the overall conformation in neutral solution of BSA is very similar to the heart shaped structure observed in the HSA crystal [12]. This is what we now see in detail: the 16-DSA probed surface of BSA mirrors the crystal structure of HSA. In contrast, we show the solution structure of HSA probed by 16-DSA is different than its crystal structure.

Taking into account that drying transition zones occur next to hydrophobic residues [7], the pronounced conformational flexibility on the surface of HSA may well be correlated with lack of energetically favorable water interactions. This is quantified and mirrored by an excess net hydropathy sum value of 48.4 (Ω_KD,HSA_ - Ω_KD,BSA_) compared to BSA, which means that HSA is severely less hydrophilic. Note that locally, many of these overall different, more hydrophobic, sites in HSA are explicitly water exposed. One may speculate that by exposing more hydrophobic amino acids, HSA may be able to better attract and pre-bind hydrophobic or amphiphilic ligands such as FAs. Furthermore, having less individual favorable interactions with water (see Fig. 6 and 7) at decisive points or short sequences and being less well incorporated in the water H-bonding network may be seen as a decrease in overall protein tertiary structural stability, which may force HSA to sample a larger conformational space and to locally feature much increased flexibility. According to the LCW-Theory [7], flexibility in our terms can also be described as a water density and thus a viscosity decrease at hydrophobic surfaces, which of course leads to an increased mean square displacement of hydrophobic residues that may e.g. be described by the simple and well known Stokes-Einstein equation [55]. All these local changes may then globally add up to a solution structure that severely deviates from the according crystal structure and which may even be considered to be specialized for different functional abilities. In the case of albumins shown in this study, these functional features are formation, location, affinity, and flexibility of binding sites for low-polarity/hydrophilicity ligands such as FAs.

### Conclusions

We have presented experimental evidence for the significant effect that water can have on the functional structure of proteins in solution. We focus on human and bovine serum albumin as model proteins which have an amino acid sequence identity of 75.52%. Our DEER-derived structures of HSA (reported previously) and BSA (reported here) loaded with FAs in solution globally characterize the tertiary protein structure from the bound ligands’ points of view. The solution structures complement the primary structures and crystal structures of HSA and as of recently also BSA. We show that the characteristic asymmetric FA distribution in the crystal structure of HSA can surprisingly be observed by DEER in BSA solution. This indicates that the BSA conformational ensemble in solution seems to be closely related to the crystal structure and hence less flexible in comparison to HSA, where a much more symmetric FA distribution was found. Conformational adaptability and flexibility of proteins can be verified on the surface of the HSA solution structure as probed with 16-DSA. This is in line with the proposition by Heidorn and Trewhella [28], that a conformational rearrangement occurs when a protein is exposed to water. We here show, to the best of our knowledge for the first time, that BSA largely lacks the conformational flexibility observed in HSA, and that water does not behave linearly on the proteins surface, but builds up clusters of varying dynamical properties as shown by Nucci et al. [10] by NMR. We further show that differences in amino acid hydropathies are not homogeneously distributed but are clustered in specific structural regions. We have identified four regions that may very strongly influence the FA binding (binding sites 1 and 5) or even the global structure (central interface between subdomains IB and IIIA and loop regions in subdomain IIB). While we cannot directly, one-to-one, link the observed difference in the solution structure topology to the differences on the primary structure level, it can be made plausible that a few amino acid differences at strategically important points can lead to the observed differences. This e.g. involves having more water molecules penetrating deeper in an interfacial region for HSA or having a strongly water exposed loop region with a much higher hydrophobicity in HSA. Such, we provide evidence that with a simple and straightforward measure like the hydropathy index it is possible to approximate possible effects on tertiary structure that can arise from protein water interactions in general. For a more detailed view concerning the specific effects of hydrophobic regions one may have to use solution-NMR based methods as NOE/ROE ratios, possibly of individual protein fragments (as previously used for HSA by Bhattacharya et al. [27]. Advanced MD simulation approaches, potentially employing the 3-dimensional molecular hydrophobicity potential (MHP) [31], may also be able to reveal allosteric conformational changes that are derived from differences in water-amino acid interactions between BSA and HSA. Furthermore, using our approach for studying other mammalian albumins (e.g. of monkeys) with amino acid sequence identities intermediate between BSA and HSA may be very insightful.

Given the often encountered view that BSA and HSA may well be interchanged and treated as if they are identical, the apparent functional structure differences should in future studies be taken into account thoroughly. Our findings indicate that directly relating crystal structures with solution structures and finally with physiological function may not always be a valid approach and in particular a re-evaluation of the hydrophobic effect as proposed by Cooper [56] could lead to a better understanding of solution structure-function relationships.

## Supporting Information

Figure S1 **Chemical structures of paramagnetic and diamagnetic fatty acids.** (*A*) Chemical structures of the paramagnetic fatty acids, 16-DSA and 5-DSA. (*B*) Reduction of the paramagnetic 16-DSA into diamagnetic fatty acid (rDSA).
(TIF)

Figure S2 **CW EPR measurements in HSA and BSA.** CW EPR spectra of (*A*) 5-DSA and (*B*) 16-DSA in HSA (red) and in BSA (black) with different albumin:DSA ratios recorded at 298 K. The characteristic signatures of DSA bound to albumin are marked by solid lines. ΔB marks the spectral separation of the outer extrema.
(TIF)

Figure S3 **CW EPR measurements in BSA with 5- and 16-DSA.** CW EPR spectra of (*A*) 5-DSA and (*B*) 16-DSA in BSA with different albumin:DSA:rDSA ratios recorded at 298 K.
(TIF)

Figure S4 **3-D view of amino acid differences between HSA and BSA.** HSA (blue, pdb-ID: 1e7i) with different amino acids compared to BSA highlighted in red (*A*) front view (*B*) 180° turned around z-axis (*C*) −90° turned around x-axis (*D*) +90° turned around x-axis.
(TIF)

Figure S5 **Amino acid sequence and hydropathy alignment of HSA and BSA (1).** Alignment of amino acids in HSA (left) and in BSA (right). (*A*) for residues 180–200 and for 453–461 located between subdomains IB and IIIA, (*B*) for residues 118–137 and for 155–167 at site 1, and (*C*) for residues 500–507 and for 559–582 at site 5. All values given are from the Kyte & Doolittle scale.
(TIF)

Figure S6 **Amino acid sequence and hydropathy alignment of HSA and BSA (2).** Alignment of amino acids in HSA (left) and in BSA (right) for residues 293–323 and for 351–380 located in subdomain IIB.
(TIF)

Figure S7 **Comparison of normalized hydropathy scales of independent origin.** Kyte & Doolittle [42]-normalized hydropathy scales: GES [39], ES [40] and NM [41]. In brackets: AAindex-ID (http://www.genome.jp)
(TIF)

Table S1 **Pearsońs r analysis.** Cross correlations (“Pearson r” values) of different hydropathy scales as calculated from the AAindex homepage.
(TIF)

Table S2 **Original and normalized hydropathy values.** Hydropathy values of all four hydropathy scales. Three (GES, ES and NM) were renormalized to the KD (Kyte & Doolittle) scale (*) so that they are directly comparable with the the KD scale.
(TIF)

Table S3 **RMSD analysis.** Imposing a complete list of RMSD values determined from Fig. 3A and 3B with an appropriate color code.
(TIF)

Supporting Information S1 (DOC)

## References

[1] Ben-Naim A (1994) Solvation: from small to macro molecules. A Curr Opin Struct Biol 4: 264–268.

[2] Kirkwood JG (1954) The General Theory of Irreversible Processes in Solutions of Macromolecules. J Polym Sci 12: 1–14.

[3] Kauzmann W (1959) Some factors in the Interpretation of protein denaturation. Adv Prot Chem 14: 1–64. 10.1016/s0065-3233(08)60608-7 14404936

[4] Ben-Naim A, Navarro AM, Leal JM (2008) A Kirkwood-Buff analysis of local properties of solutions. Phys Chem Chem Phys 10: 2451–2460. 18446245 10.1039/b716116f

[5] Cooper A (1976) Thermodynamic fluctuations in protein molecules. Proc Natl Acad Sci USA 73: 2740–2741. 1066687 10.1073/pnas.73.8.2740 PMC430724

[6] Patel AJ, Varilly P, Jamadagni SN, Hagan MF, Chandler D, et al (2012) Sitting at the Edge: How Biomolecules use Hydrophobicity to Tune Their Interactions and Function. Phys Chem B 116: 2498–2503. 10.1021/jp2107523 PMC3303187 22235927

[7] Lum K, Chandler D, Weeks JD (1999) Hydrophobicity at Small and Large Length Scales. J Phys Chem B 103: 4570–4577.

[8] Ben-Naim A (2006) On the driving forces for protein-protein association. J Chem Phys 125: 024901. 10.1063/1.2205860 16848605

[9] Ben-Naim A (2008) One-dimensional model for water and aqueous solutions. IV. A study of “hydrophobic interactions”. J Chem Phys 129: 104506. 19044923 10.1063/1.2976442

[10] Nucci NV, Pometun MS, Wand AJ (2011) Site-resolved measurement of water-protein interactions by solution NMR. Nat Struct Mol Biol 18: 245–250. 21196937 10.1038/nsmb.1955 PMC3058360

[11] Peters T (1995) All about albumin: biochemistry, genetics and medical applications. San Diego: Academic Press. 432p.

[12] Ferrer ML, Duchowicz R, Carrasco B, Garcia de la Torre J, Acuna AU (2001) The Conformation of Serum Albumin in Solution: A Combined Phosphorescence Depolarization-Hydrodynamic Modeling Study. Biophys J 80: 2422–2430. 11325741 10.1016/S0006-3495(01)76211-X PMC1301430

[13] Tanford C, Buzzell JG (1956) The viscosity of aqueous solutions of Bovine Serum Albumin between pH 4.3 and 10.5. J Phys Chem 60: 225–231.

[14] Carter DC, He JX (1990) Structure of human serum albumin. Science 249: 302–304. 2374930 10.1126/science.2374930

[15] Trivedi VD, Saxena I, Siddiqui MU, Qasim MA (1997) Interaction of bromocresol green with different serum albumins studied by fluorescence quenching. Biochem Mol Biol Int 43: 1–8. 9315276 10.1080/15216549700203751

[16] Junk MJN, Spiess HW, Hinderberger D (2010) The Distribution of Fatty Acids Reveals the Functional Structure of Human Serum Albumin. Angew Chemie Int Ed 49: 8755–8759. 10.1002/anie.201003495 20886483

[17] Akdogan Y, Junk MJN, Hinderberger D (2011) Effect of Ionic Liquids on the Solution Structure of Human Serum Albumin. Biomacromolecules 12: 1072–1079. 21332184 10.1021/bm1014156

[18] Junk MJN, Spiess HW, Hinderberger D (2011) DEER in biological multispin-systems: A case study on the fatty acid binding to human serum albumin. J Magn Reson 210: 210–217. 21450500 10.1016/j.jmr.2011.03.003

[19] Akdogan Y, Hinderberger D (2011) Solvent-Induced Protein Refolding at Low Temperatures. J Phys Chem B 115: 15422–15429. 22114991 10.1021/jp209646f

[20] Junk MJN, Spiess HW, Hinderberger D (2011) Characterization of the Solution Structure of Human Serum Albumin Loaded with a Metal Porphyrin and Fatty Acids. Biophys J 100: 2293–2301. 21539799 10.1016/j.bpj.2011.03.050 PMC3149252

[21] Hubbell WL, Altenbach C (1994) Investigation of structure and dynamics in membrane proteins using site-directed spin labeling. Curr Opin Struct Biol 4: 566–573.

[22] Hubbell WL, Cafiso D, Altenbach C (2000) Identifying conformational changes with site-directed spin labeling. Nature Struct Biol 7: 735–739. 10966640 10.1038/78956

[23] Georgieva ER, Ramlall TF, Borbat PP, Freed JH, Eliezer D (2010) The Lipid-binding Domain of Wild Type and Mutant α-Synuclein. J Biochem Chem 285: 28261–28274. 10.1074/jbc.M110.157214 PMC2934691 20592036

[24] Hänelt I, Wunnicke D, Müller-Trimbusch M, Vor der Brüggen M, Kraus I, et al (2010) Membrane Region M_2C2_ in Subunit KtrB of the K^+^ uptake System KtrAB from *Vibrio alginolyticus* Forms a Flexible Gate Controlling K^+^ Flux. J Biol Chem 285: 28210–28219. 20573964 10.1074/jbc.M110.139311 PMC2934686

[25] Bird GH, Pornsuwan S, Saxena S, Schafmeister CE (2008) Distance Distributions of End-Labeled Curved Bispeptide Oligomers by Electron Spin Resonance. ACS Nano 2: 1857–1864. 19206425 10.1021/nn800327g

[26] Jeschke G, Chechik V, Ionita P, Godt A, Zimmermann H, et al (2006) DeerAnalysis2006– a comprehensive software package for analyzing pulsed ELDOR data. Appl Magn Reson 30: 473–498.

[27] Bhattacharya AA, Grüne T, Curry S (2000) Crystallographic Analysis Reveals Common Modes of Binding of Medium and Long-chain Fatty Acids to Human Serum Albumin. J Mol Biol 303: 721–732. 11061971 10.1006/jmbi.2000.4158

[28] Heidorn DB, Trewhella J (1988) Comparison of the Crystal and Solution Structures of Calmodulin and Troponin C. Biochemistry. 27: 909–915. 10.1021/bi00403a011 3365370

[29] Chen H, Deere M, Hecht JT, Lawler JJ (2000) Cartilage Oligomeric Matrix Protein Is a Calcium-binding Protein, and a Mutation in Its Type 3 Repeats Causes Conformational Changes. J Biol Chem 275: 26538–26544. 10852928 10.1074/jbc.M909780199

[30] Watkins S, Sakamoto Y, Madison J, Davis E, Smith DG, et al (1993) cDNA and protein sequence of polymorphic macaque albumins that differ in bilirubin binding. Proc Natl Acad Sci USA 90: 2409–2413. 8460152 10.1073/pnas.90.6.2409 PMC46096

[31] Efremov RG, Chugunov AO, Pyrkov TV, Priestle JP, Arseniev AS, et al (2007) Molecular Lipophilicity in Protein Modeling and Drug Design. Curr Med Chem 14: 393–415. 17305542 10.2174/092986707779941050

[32] Polyansky AA, Zagrovic B (2012) Protein Electrostatic Properties Predefining the Level of Surface Hydrophobicity Change upon Phosphorylation. J Phys Chem Lett 3: 973–976. 23914287 10.1021/jz300103p PMC3726239

[33] Krieger E, Darden T, Nabuurs SB, Finkelstein A, Vriend G (2004) Making optimal use of empirical energy functions: Force-field parametrization in crystal space. Proteins 57: 678–683. 15390263 10.1002/prot.20251

[34] Jeschke G, Sajid M, Schulte M, Godt A (2009) Three-spin correlations in double electron-electron resonance. Phys Chem Chem Phys 11: 6580–6591. 19639133 10.1039/b905724b

[35] Majorek KA, Porebski PJ, Dayal A, Zimmerman MD, Jablonska K, Stewart AJ, Chruszcz M, Minor W (2012) Structural an immunologic characterization of bovine, horse, and rabbit serum albumins. Molecular Immunology 52: 174–182. 22677715 10.1016/j.molimm.2012.05.011 PMC3401331

[36] Sugio S, Kashima A, Mochizuki S, Noda M, Kobayashi K (1999) Crystal Structure of human serum albumin at 2.5 Å resolution. Protein Eng 12: 439–446. 10388840 10.1093/protein/12.6.439

[37] Konagurthu AS, Whisstock JC, Stuckey PJ, Lesk AM (2006) MUSTANG: A Multiple Structural Alignment Algorithm. Proteins: Structure, Function, and Bioinformatics 64: 559–574. 10.1002/prot.20921 16736488

[38] Huang DM, Chandler D (2000) Temperature and length scale dependence of hydrophobic effects and their possible implications for protein folding. Proc Natl Acad Sci USA 97: 8324–8327. 10890881 10.1073/pnas.120176397 PMC26946

[39] Engelman DM, Steitz TA, Goldman A (1986) Identifying nonpolar transbilayer helices in amino acid sequences of membrane proteins. Ann Rev Biophys Biophys Chem 15: 321–53. 3521657 10.1146/annurev.bb.15.060186.001541

[40] Eisenberg D, Schwarz E, Komaromy M, Wall R (1984) Analysis of Membrane and Surface Protein Sequences with the Hydrophobic Moment Plot. J Mol Biol 179: 125–142. 6502707 10.1016/0022-2836(84)90309-7

[41] Naderi-Manesh H, Sadeghi M, Arab S, Movahedi AAM (2001) Prediction of Protein Surface Accessibility with Information Theory. Proteins 42: 452–459. 11170200 10.1002/1097-0134(20010301)42:4<452::aid-prot40>3.0.co;2-q

[42] Kyte J, Doolittle RF (1982) A Simple Method for Displaying the Hydropathic Character of a Protein. J Mol Biol 157: 105–132. 7108955 10.1016/0022-2836(82)90515-0

[43] Nelson D, Cox M, Lehninger AL (2009) Biochemie. 4th edition, Springer-Verlag. 1712p.

[44] Billeter M, Riek R, Wider G, Hornemann S, Glockshuber R, et al (1997) Prion protein NMR structure and species barrier for prion diseases. Proc Natl Acad Sci USA. 94: 7281–7285. 10.1073/pnas.94.14.7281 PMC23812 9207082

[45] Simard JR, Zunszain PA, Ha CE, Yang JS, Bhagavan NV, Petitpas I, Curry S, Hamilton JA (2005) Locating high-affinity fatty acid-binding sites on albumin by x-ray crystallography and NMR spectroscopy. PNAS 102: 17958–17963. 16330771 10.1073/pnas.0506440102 PMC1312385

[46] Kabsch W, Sander C (1984) On the use of sequence homologies to predict protein structure: Identical pentapeptides can have completely different conformations. Proc Natl Acad Sci USA 81: 1075–1078. 6422466 10.1073/pnas.81.4.1075 PMC344767

[47] Kim M, Xu Q, Murray D, Cafiso DS (2008) Solutes Alter the Conformation of the Ligand Binding Loops in Outer Membrane Transporters. Biochemistry 47: 670–679. 18092811 10.1021/bi7016415

[48] Marlow MS, Dogan J, Frederick KK, Valentine KG, Wand AJ (2010) The role of conformational entropy in molecular recognition by calmodulin. Nat Chem Biol 6: 352–358. 20383153 10.1038/nchembio.347 PMC3050676

[49] Berg JM, Tymoczko JL, Stryer L (2011) Biochemie. 6th edition, Spektrum Akademischer Verlag. 1264p.

[50] Heller J, Elgabarty H, Zhuang B, Sebastiani D, Hinderberger D (2010) Solvation of Small Disulfonate Anions in Water/Methanol Mixtures Characterized by High-Field Pulse Electron Nuclear Double Resonance and Molecular Dynamics Simulations. J Phys Chem B 114: 7429–7438. 20465252 10.1021/jp910335t

[51] Sander C, Schneider R (1991) Database of Homology-Derived Protein Structures and the Structural Meaning of Sequence Alignment. Proteins 9: 56–68. 2017436 10.1002/prot.340090107

[52] Booth DR, Sunde M, Bellotti V, Robinson CV, Hutchinson WL, et al (1997) Instability, unfolding and aggregation of human lysozyme variants underlying amyloid fibrillogenesis. Nature 385: 787–793. 9039909 10.1038/385787a0

[53] Dobson CM (2003) Protein folding and misfolding. Nature 426: 884–890. 14685248 10.1038/nature02261

[54] Inouye H, Bond J, Baldwin MA, Ball HL, Prusiner SB (2000) Structural Changes in a Hydrophobic Domain of the Prion Protein Induced by Hydration and by Ala Val and Pro Leu Substitutions. J Mol Biol 300: 1283–1296. 10903869 10.1006/jmbi.2000.3926

[55] Atkins PW (2001) Physikalische Chemie. 3rd edition. Wiley-VCH. 1106p.

[56] Cooper A (2005) Heat capacity effects in protein folding and ligand binding: a re-evaluation of the role of water in biomolecular thermodynamics. Biophys Chem 115: 89–97. 15752588 10.1016/j.bpc.2004.12.011

