# Supplementary figures and images for: Evidence for Water-Tuned Structural Differences in Proteins: An Approach Emphasizing Variations in Local Hydrophilicity
Source: PLoS One. 2012 Sep 25;7(9):e45681. doi: 10.1371/journal.pone.0045681 (PMC3458090; doi:10.1371/journal.pone.0045681)

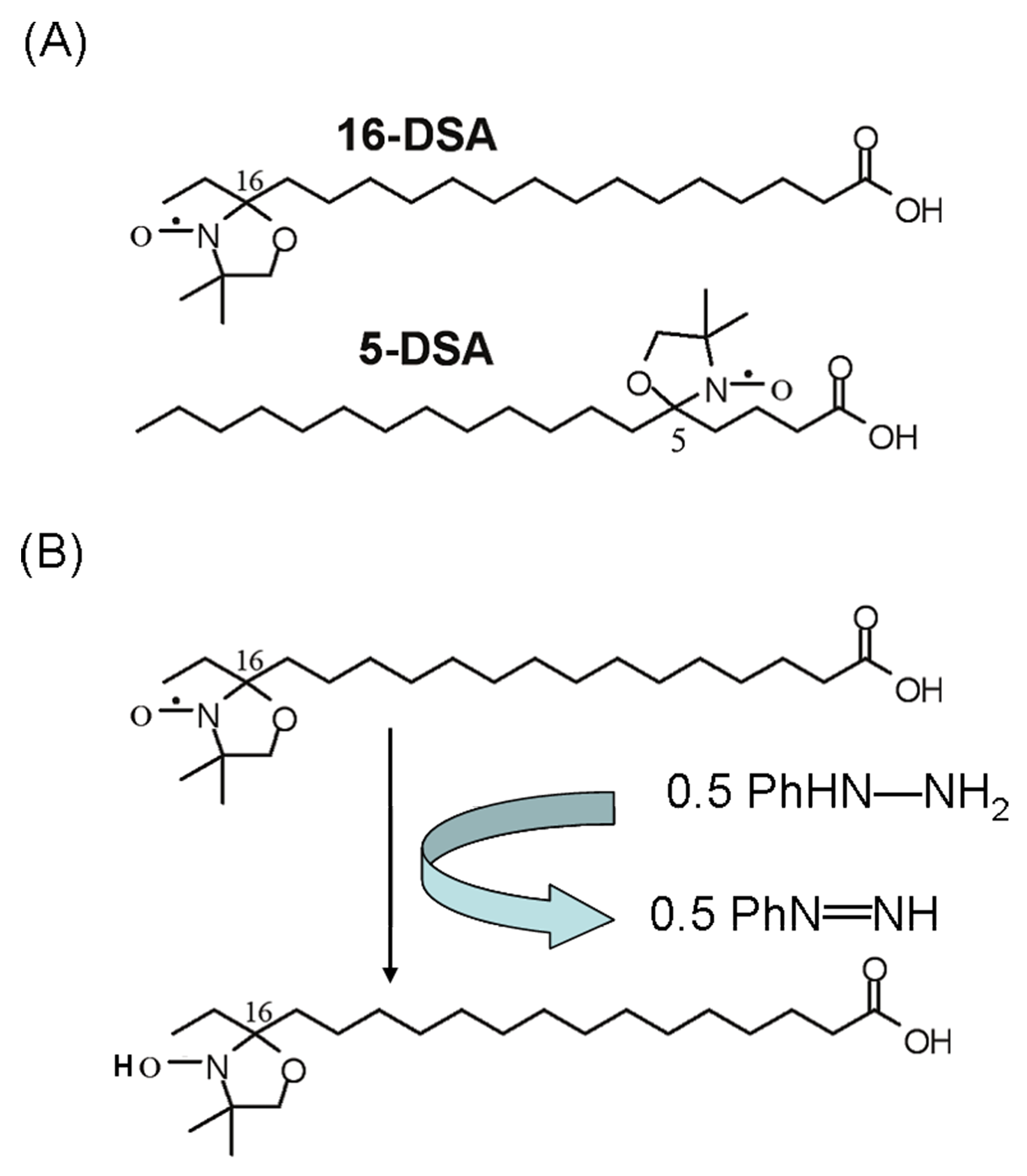

Supplement: Figure S1 — Chemical structures of paramagnetic and diamagnetic fatty acids. (A) Chemical structures of the paramagnetic fatty acids, 16-DSA and 5-DSA. (B) Reduction of the paramagnetic 16-DSA into diamagnetic fatty acid (rDSA). (TIF) [file pone.0045681.s001.tif]

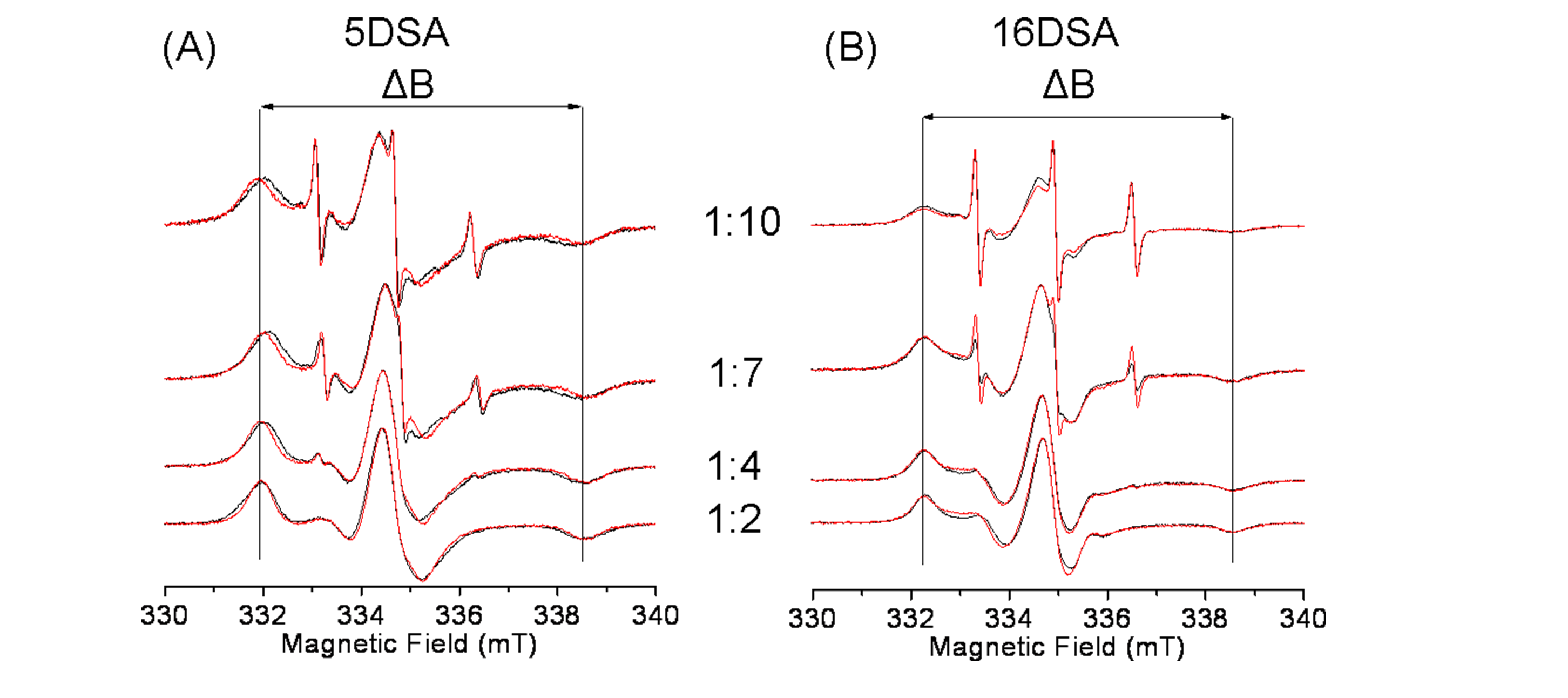

Supplement: Figure S2 — CW EPR measurements in HSA and BSA. CW EPR spectra of (A) 5-DSA and (B) 16-DSA in HSA (red) and in BSA (black) with different albumin:DSA ratios recorded at 298 K. The characteristic signatures of DSA bound to albumin are marked by solid lines. ΔB marks the spectral separation of the outer extrema. (TIF) [file pone.0045681.s002.tif]

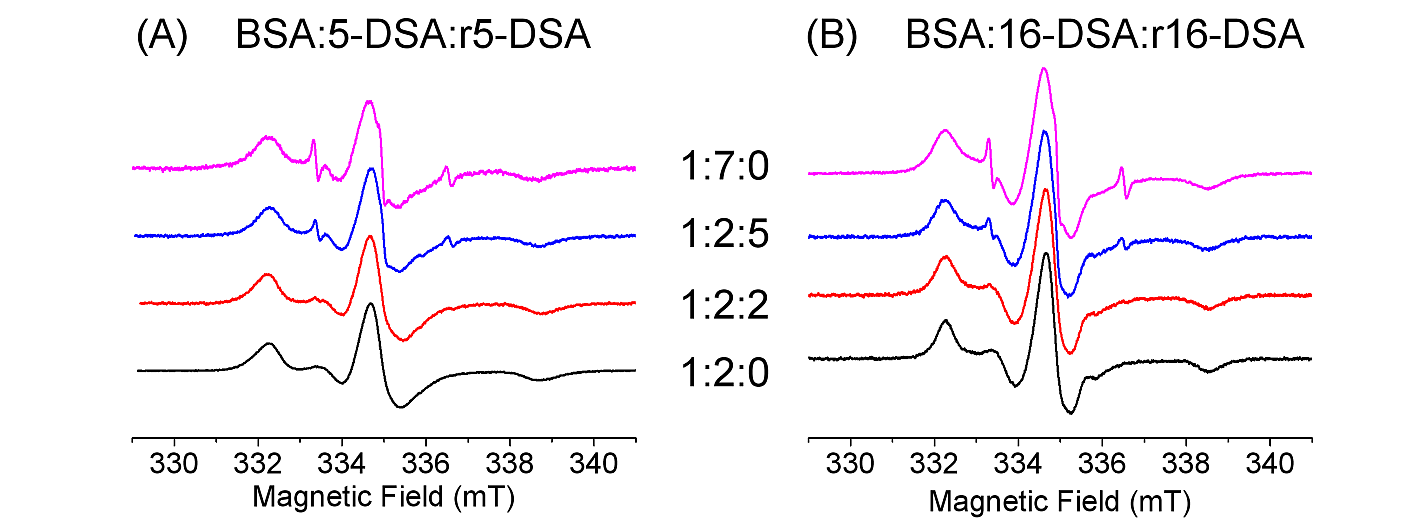

Supplement: Figure S3 — CW EPR measurements in BSA with 5- and 16-DSA. CW EPR spectra of (A) 5-DSA and (B) 16-DSA in BSA with different albumin:DSA:rDSA ratios recorded at 298 K. (TIF) [file pone.0045681.s003.tif]

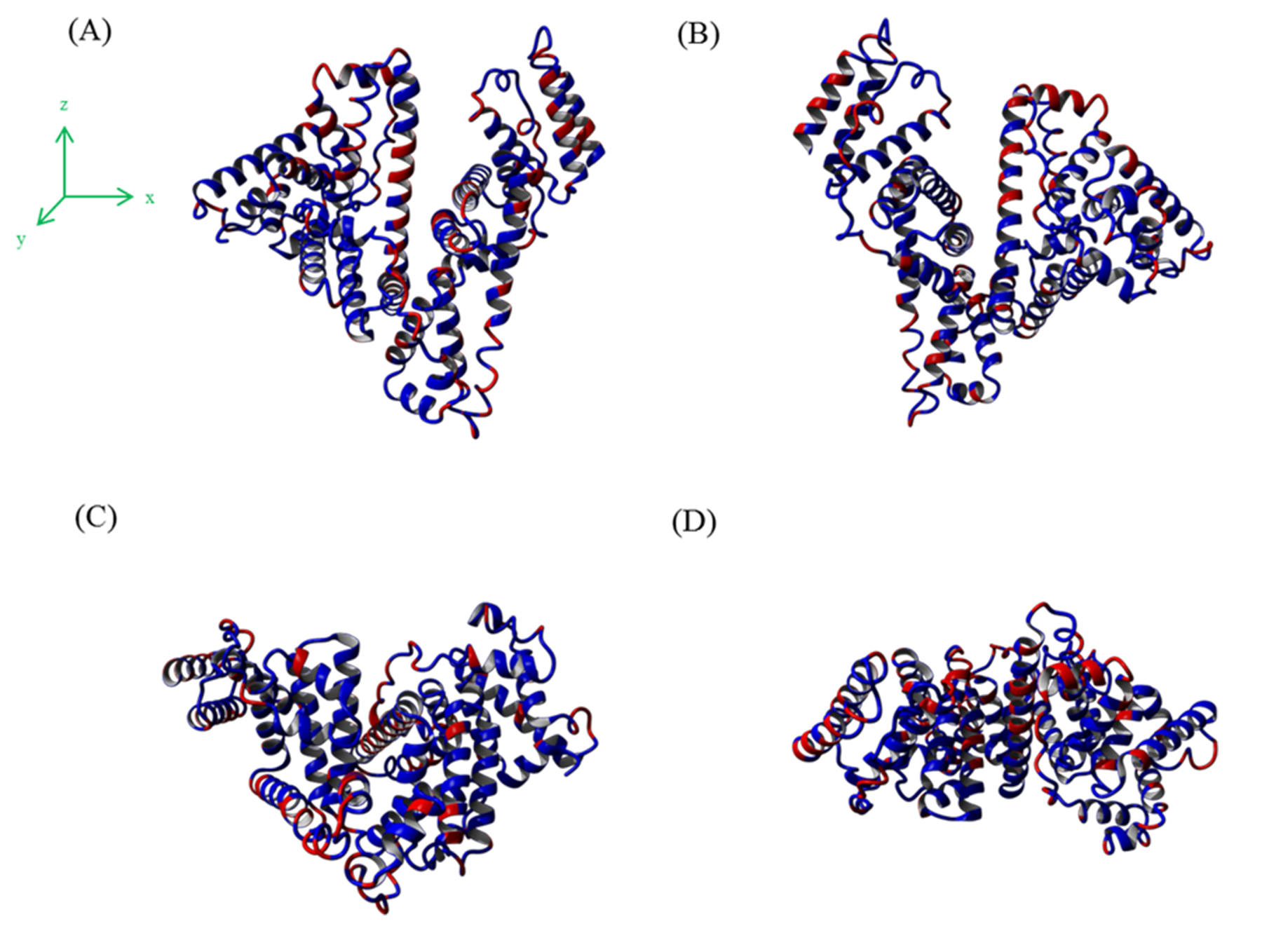

Supplement: Figure S4 — 3-D view of amino acid differences between HSA and BSA. HSA (blue, pdb-ID: 1e7i) with different amino acids compared to BSA highlighted in red (A) front view (B) 180° turned around z-axis (C) −90° turned around x-axis (D) +90° turned around x-axis. (TIF) [file pone.0045681.s004.tif]

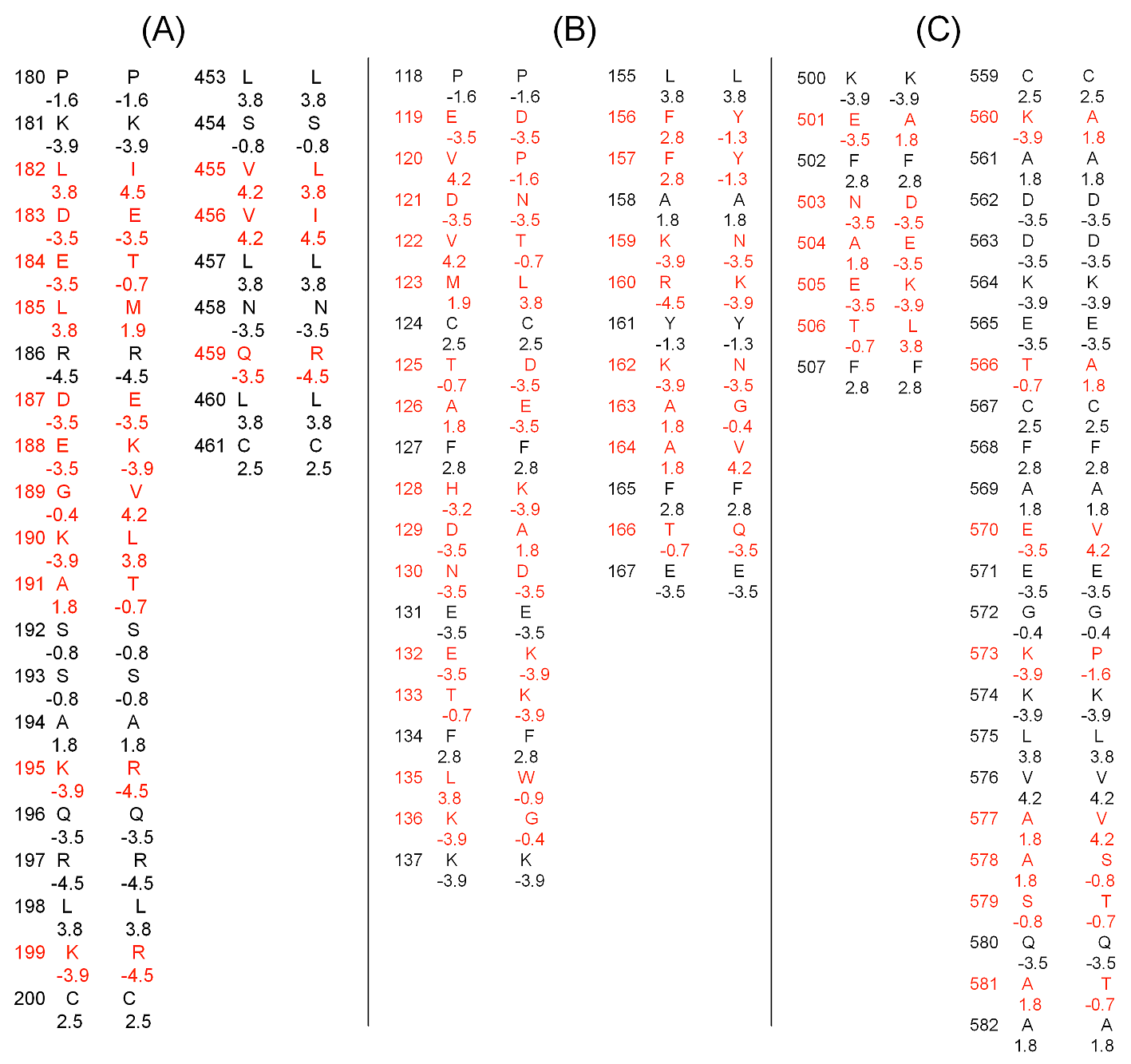

Supplement: Figure S5 — Amino acid sequence and hydropathy alignment of HSA and BSA (1). Alignment of amino acids in HSA (left) and in BSA (right). (A) for residues 180–200 and for 453–461 located between subdomains IB and IIIA, (B) for residues 118–137 and for 155–167 at site 1, and (C) for residues 500–507 and for 559–582 at site 5. All values given are from the Kyte & Doolittle scale. (TIF) [file pone.0045681.s005.tif]

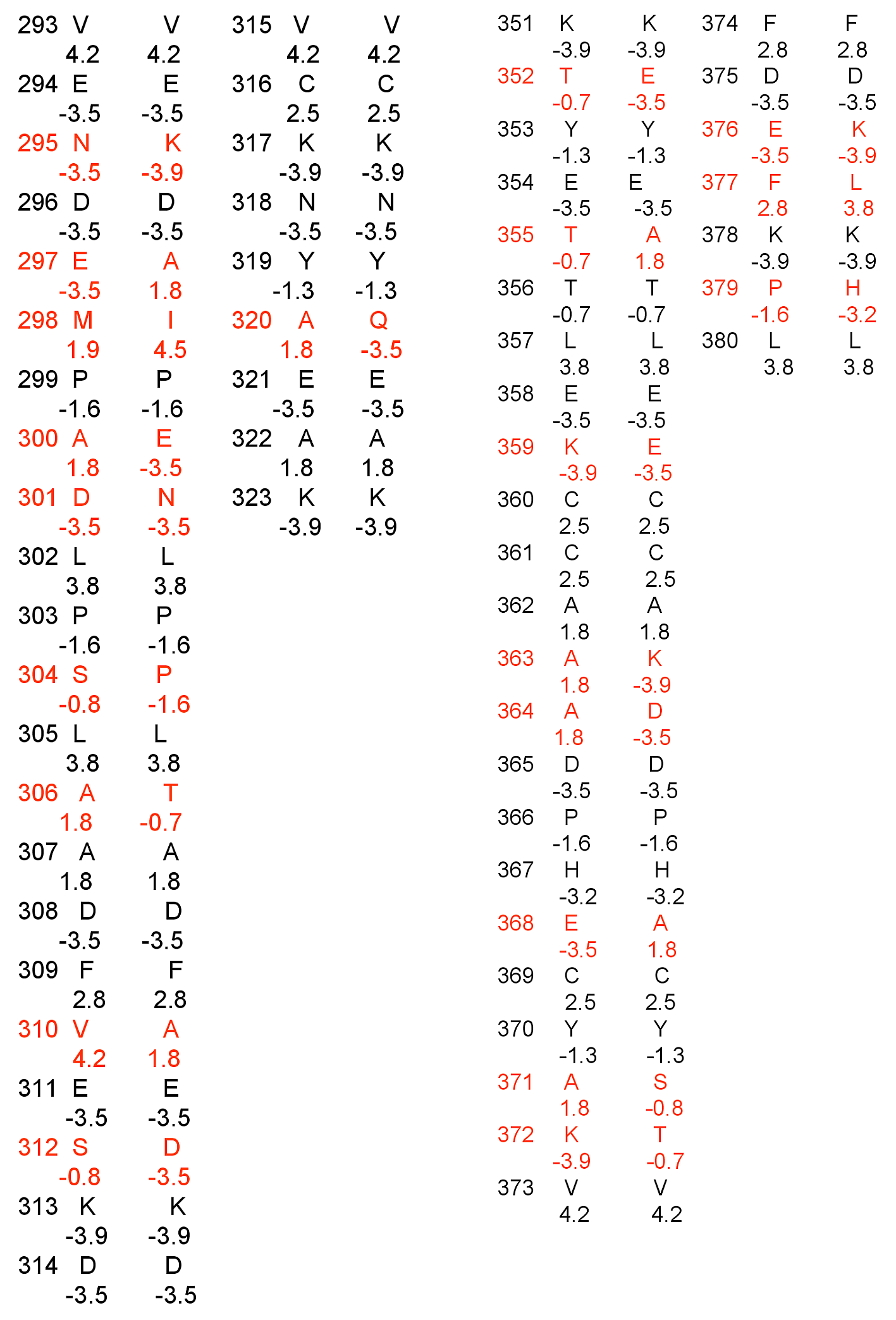

Supplement: Figure S6 — Amino acid sequence and hydropathy alignment of HSA and BSA (2). Alignment of amino acids in HSA (left) and in BSA (right) for residues 293–323 and for 351–380 located in subdomain IIB. (TIF) [file pone.0045681.s006.tif]

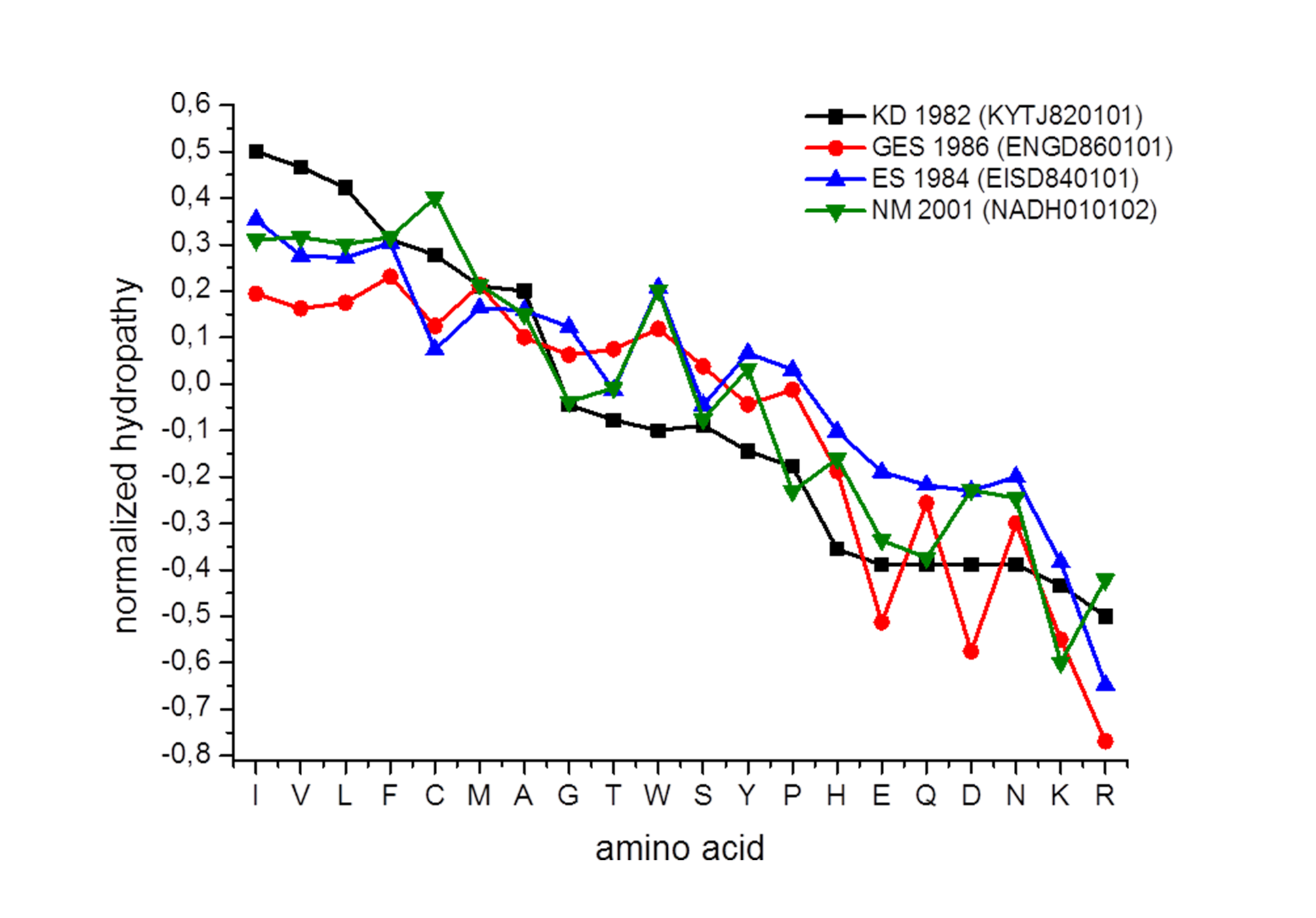

Supplement: Figure S7 — Comparison of normalized hydropathy scales of independent origin. Kyte & Doolittle [42]-normalized hydropathy scales: GES [39], ES [40] and NM [41]. In brackets: AAindex-ID (http://www.genome.jp) (TIF) [file pone.0045681.s007.tif]

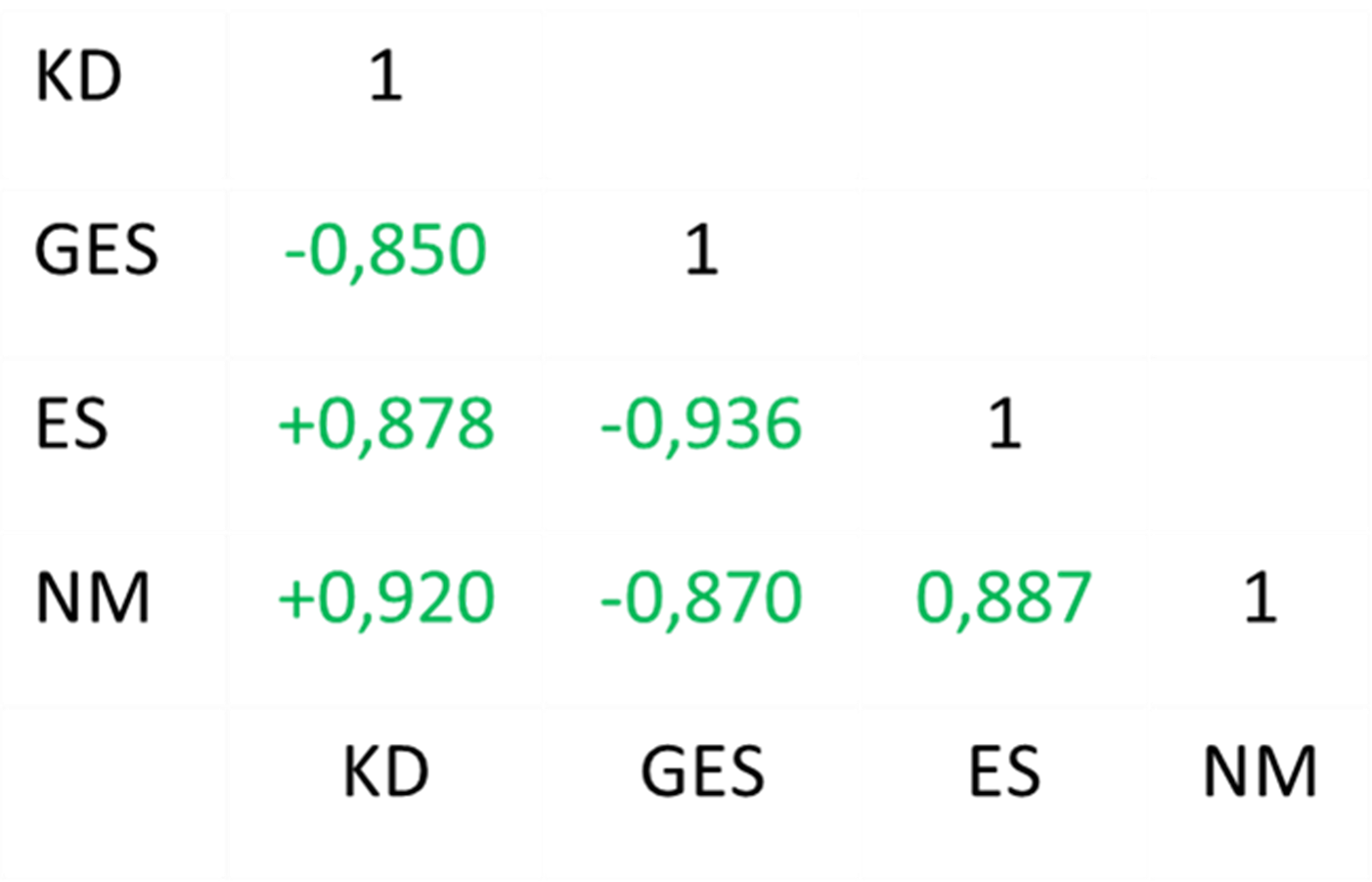

Supplement: Table S1 — Pearsońs r analysis. Cross correlations (“Pearson r” values) of different hydropathy scales as calculated from the AAindex homepage. (TIF) [file pone.0045681.s008.tif]

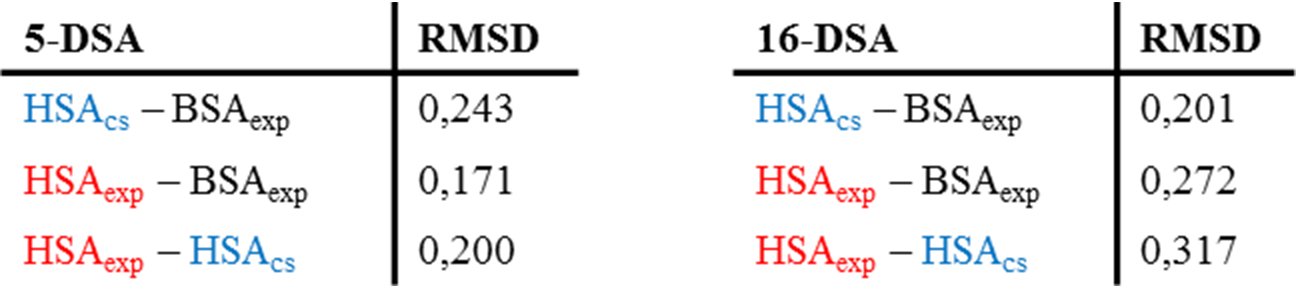

Supplement: Table S3 — RMSD analysis. Imposing a complete list of RMSD values determined from Fig. 3A and 3B with an appropriate color code. (TIF) [file pone.0045681.s010.tif]
