# Supplementary material for: Evidence for Water-Tuned Structural Differences in Proteins: An Approach Emphasizing Variations in Local Hydrophilicity
Source: PLoS One. 2012 Sep 25;7(9):e45681. doi: 10.1371/journal.pone.0045681 (PMC3458090; doi:10.1371/journal.pone.0045681)
Supplement: Supporting Information S1 — (DOC) [file pone.0045681.s011.doc]

Evidence for water-tuned structural differences in proteins: An approach emphasizing variations in local hydrophilicity

Yasar Akdogan^§,&^, Jörg Reichenwallner^§,&,#^, Dariush Hinderberger^§^,*

^§^Max Planck Institute for Polymer Research, Mainz, Germany

^#^Institut für Pharmazie und Biochemie, Johannes Gutenberg-Universität Mainz, Mainz, Germany

^&^ These authors contributed equally

^*^Address correspondence to: [dariush.hinderberger@mpip-mainz.mpg.de](mailto:dariush.hinderberger@mpip-mainz.mpg.de)

SUPPORTING INFORMATION

**CW EPR measurements and further discussions on DEER-timetraces**

The occupation of different binding pockets in HSA and BSA by DSA and rDSA (Fig. S1) can be monitored by CW EPR measurements as shown in Fig. S2 and Fig. S3.

Fig. 2 of the manuscript also shows the intramolecular parts of the DEER time domain data and extracted distance distributions when BSA is loaded with exclusively seven paramagnetic 5-DSA or 16-DSA. As expected, the modulation depths Δ of these systems are much larger than of those obtained with spin-diluted BSA-fatty acid systems as the modulation depth is a measure of the number of interacting spins [1,2]. The conversion of the time domain data of systems with more than two spins into distance distributions causes artifacts such as broadening of the distance peaks, overestimation of small distances and suppression of large distances. Hence, the seven paramagnetic FA-BSA system has a distance distribution with strongly broadened distance peaks and an increased population of short distances compared to the results of spin-diluted BSA-fatty acid (1:2:5) systems (also shown in Fig. 2 *C* and *D*).

**A comparative view on HSA and BSA crystal structures**

All ribbon style illustrations have been established using YASARA Structure software [3]. For simplification, the comparison between HSA and BSA was carried out in the framework of HSA with seven FAs bound (pdb-ID: 1e7i) [4] to have a functional view on the crystal structure. HSA backbone matrix was kept in blue, whereas differences to BSA in amino acid sequence were highlighted in red (Fig. S4). For alignment of the amino acids, we gained the according sequences in FASTA-format (1BM0 [5] and 3v03 [6]) from the RCSB homepage (see also Fig. S5 and Fig. S6). At residue position 116, BSA was found to lack a Valin which shifts the whole working frame of this protein for one amino acid. The original BSA sequence positions exceeding residue 116 are thus (i-1) compared to HSA.

**3D-Hydropathy analysis of HSA and BSA**

The hydropathy values in Fig. S5 and Fig. S6 were taken from the hydropathy scale of Kyte and Doolittle [7].

Unlike their SOAP program which can discriminate hydrophobic and hydrophilic regions in proteins by applying different sliding average window ranges, we chose a window range of one residue to get rid of generalizations and difficult decisions which have to be made with this approach. The Δhydropathy (ΔHI) values are determined due to:

$\Delta HI_{i}=HI_{HSA,i}-HI_{BSA,i}$ (Eq. S1)

And the overall hydropathies:

$\Omega_{x}=\sum_{i}^{N(x)} HI_{x,i}$ (Eq. S2)

where *x* can be an arbitrary polypeptide chain of length *N(x)* of a protein *x* and *i* is the respective amino acid according to its chain position, disregarding surface exposition. Histograms below each following figure illustrate the difference of the corresponding amino acid values from and additionally we get rid of the common noisy lines appearing at low window ranges. Furthermore, when not explicitly mentioned, the amino acids are kept in the common one letter code.

**Comparison of Kyte & Doolittle with other hydropathy scales**

We carried out a comparison of four different normalized hydropathy scales of completely independent origin for consistency check.

**1) KD:** Kyte & Doolittle [7]: based on water-vapor-transfer energies and interior/

exterior amino acid distributions.

**2) GES:** Engelman et al. [8]: amino acid free energy transfer from water to oil,

including hydrophobic (surface area calculations) and hydrophilic (hydrogen

bonding, pKa) considerations.

**3) ES:** Eisenberg et al. [9]: a normalized consensus scale, calculated from hydro-

phobic dipole moments, based on 5 other scales.

**4) NM:** Naderi-Manesh et al. [10]: using information theory to predict solvent accessi-

bility from the propensity of amino acids to take over certain conformations,

depending on according local environment.

A first quantitative estimate of correlations between different hydropathy scales is given by the “Pearson´s r” value. It delivers a number for cross correlations and linear depen- dences. For strong correlations “Pearson´s r” has to be:

$$\text{Pearson r}\in\left\{\text{ |}\pm\text{1| }\leq\text{x}\leq\text{ }|\pm0,5|\text{ }\right\}$$

Assigning those values to our above mentioned scales we get the corresponding numbers in Table S1.

As we can see, each hydropathy scale in use is strongly correlated with the others, although having different theoretical and experimental foundations.

In Figure S7 the normalized hydropathy values from Table S2 are set out graphically and show intriguing similarity. Furthermore, Figures 5-8 show the hydropathy difference (ΔHI) between HSA and BSA for four chosen regions in the proteins. There, we renormalized each scale to KD values (value range [-4.5; 4.5]).

Value ranges (*scaled to KD): GES*: [-6,919; 2,081]

ES*: [ 3,176; -5,824]

NM*: [ 3,605; -5,395]

The resulting excess hydropathy points ΔΩ_k_ = Ω_k,HSA_ – Ω_k,BSA_ sum up to:

ΔΩ_KD_: 48.4; ΔΩ_NM_: 34.8; ΔΩ_ES_: 16.8; ΔΩ_GES_: 9.4

Where *k* is the applied hydropathy scale.

**HSA and BSA structure alignment with MUSTANG [11]:**

Comparing pdB-IDs 1BM0 (HSA without FAs [5]) and 3v03 (BSA without FAs [6]) by alignment with MUSTANG algorithm [11], both crystal structures fit astonishingly good in 3D-topology (Fig. 4 and Fig. S4) as implemented for proteins with corresponding functions [12]. RMSD is 1.361 Å and sequence identity is 75.52 % over 572 aligned residues. Distracting atom objects, molecule objects and excess monomeric macromolecular objects of dimers were removed before alignment was started.

**Determination of RMSD values between two curves**

For a more objective view on the distance distributions of HSA and BSA, the determination of RMSD values between different curves of Fig. 3A and 3B was carried out using the processed data of the DEER timetraces, namely the distance distribution files (X_exp_) and the single distances (Fig. 1) of the minimized crystal structures read out with Jmol (X_cs_). Those 21 single distances of the latter one were artificially broadened with a homemade matlab code as in Junk et al. [13]. The broadening parameter was chosen to be: σ = 0,36.

The maximum peaks were normalized to 1 and if the datasets did not contain equal amounts of datapoints they were interpolated to the same value by the corresponding Microcal Origin tool for comparison. The distance range for comparison was kept constant at 1.5 to 8.0 nm. Each datapoint of the two curves was treated as follows to achieve the RMSD value:

(Eq. S3)

Where *y_j,i_* is the ordinate value of each of both datasets *j* = 1,2. *i* is the *i*-th datapoint of the dataset, and *n* the total number of datapoints. All calculated values are summarized in Table S3.

Supporting References

1. Junk MJN, Spiess HW, Hinderberger D (2011) DEER in biological multispin-systems: A case study on the fatty acid binding to human serum albumin. J Magn Reson 210: 210-217.
2. Jeschke G, Sajid M, Schulte M, Godt A (2009) Three-spin correlations in double electron-electron resonance. Phys Chem Chem Phys 11: 6580-6591.
3. Krieger E, Darden T, Nabuurs SB, Finkelstein A, Vriend G (2004) Making optimal use of empirical energy functions: Force-field parametrization in crystal space. Proteins 57: 678-683.
4. Bhattacharya AA, Grüne T, Curry S (2000) Crystallographic Analysis Reveals Common Modes of Binding of Medium and Long-chain Fatty Acids to Human Serum Albumin. J Mol Biol 303: 721-732.
5. Sugio S, Kashima A, Mochizuki S, Noda M, Kobayashi K (1999) Crystal Structure of human serum albumin at 2.5 Å resolution. Protein Eng 12: 439-446.
6. Majorek KA, Porebski PJ, Dayal A, Zimmerman MD, Jablonska K, Stewart AJ, Chruszcz M, Minor W (2012) Structural an immunologic characterization of bovine, horse, and rabbit serum albumins. Molecular Immunology 52: 174-182.
7. Kyte J, Doolittle RF (1982) A Simple Method for Displaying the Hydropathic Character of a Protein. J Mol Biol 157: 105-132.
8. Engelman DM, Steitz TA, Goldman A (1986) Identifying nonpolar transbilayer helices in amino acid sequences of membrane proteins. Ann Rev Biophys Biophys Chem 15: 321-53.
9. Eisenberg D, Schwarz E, Komaromy M, Wall R (1984) Analysis of Membrane and Surface Protein Sequences with the Hydrophobic Moment Plot. J Mol Biol 179: 125-142.
10. Naderi-Manesh H, Sadeghi M, Arab S, Movahedi AAM (2001) Prediction of Protein Surface Accessibility with Information Theory. Proteins: Structure, Function, and Genetics 42: 452–459.
11. Konagurthu AS, Whisstock JC, Stuckey PJ, Lesk AM (2006) MUSTANG: A Multiple Structural Alignment Algorithm. Proteins: Structure, Function, and Bioinformatics 64: 559-574.
12. Berg JM, Tymoczko JL, Stryer L (2011) Biochemie. 6th edition, Spektrum Akademischer Verlag. 1264p.
13. Junk MJN, Spiess HW, Hinderberger D (2010) The Distribution of Fatty Acids Reveals the Functional Structure of Human Serum Albumin. [Angew Chemie Int Ed 49: 8755-8759.](http://dx.doi.org/10.1002/anie.201003495)
